# Endosymbiont DNA in Endobacteria-Free Filarial Nematodes Indicates Ancient Horizontal Genetic Transfer

**DOI:** 10.1371/journal.pone.0011029

**Published:** 2010-06-09

**Authors:** Samantha N. McNulty, Jeremy M. Foster, Makedonka Mitreva, Julie C. Dunning Hotopp, John Martin, Kerstin Fischer, Bo Wu, Paul J. Davis, Sanjay Kumar, Norbert W. Brattig, Barton E. Slatko, Gary J. Weil, Peter U. Fischer

**Affiliations:** 1 Infectious Diseases Division, Department of Internal Medicine, Washington University School of Medicine, St. Louis, Missouri, United States of America; 2 New England Biolabs, Ipswich, Massachusetts, United States of America; 3 The Genome Center, Department of Genetics, Washington University School of Medicine, St. Louis, Missouri, United States of America; 4 Institute for Genome Sciences, Department of Microbiology and Immunology, University of Maryland Baltimore, Baltimore, Maryland, United States of America; 5 Bernhard Nocht Institute for Tropical Medicine, Hamburg, Germany; BMSI-A*STAR, Singapore

## Abstract

**Background:**

*Wolbachia* are among the most abundant symbiotic microbes on earth; they are present in about 66% of all insect species, some spiders, mites and crustaceans, and most filarial nematode species. Infected filarial nematodes, including many pathogens of medical and veterinary importance, depend on *Wolbachia* for proper development and survival. The mechanisms behind this interdependence are not understood. Interestingly, a minority of filarial species examined to date are naturally *Wolbachia-*free.

**Methodology/Principal Findings:**

We used 454 pyrosequencing to survey the genomes of two distantly related *Wolbachia-*free filarial species, *Acanthocheilonema viteae* and *Onchocerca flexuosa*. This screen identified 49 *Wolbachia-*like DNA sequences in *A. viteae* and 114 in *O. flexuosa*. qRT-PCR reactions detected expression of 30 *Wolbachia*-like sequences in *A. viteae* and 56 in *O. flexuosa*. Approximately half of these appear to be transcribed from pseudogenes. *In situ* hybridization showed that two of these pseudogene transcripts were specifically expressed in developing embryos and testes of both species.

**Conclusions/Significance:**

These results strongly suggest that the last common ancestor of extant filarial nematodes was infected with *Wolbachia* and that this former endosymbiont contributed to their genome evolution. Horizontally transferred *Wolbachia* DNA may explain the ability of some filarial species to live and reproduce without the endosymbiont while other species cannot.

## Introduction

Several important evolutionary milestones, such as the emergence of eukaryotes and the development of intracellular organelles (e.g. mitochondria and plastids), have involved disparate species uniting to form composite organisms. Filarial nematodes and their *Wolbachia* endobacteria are an interesting example of such a composite [1]. Although antibiotics can be used to cure arthropods of their *Wolbachia* infection, similar treatments lead to infertility, improper development and sometimes death of *Wolbachia*-dependent filarial nematodes [2], [3]. Likewise, attempts to maintain filarial *Wolbachia* in culture have failed (B.E. Slatko, P.U. Fischer, R.U. Rao, pers. comm.). This interdependence may be a consequence of reductive evolution in both partners, as several biosynthetic pathways (e.g. synthesis of heme, riboflavin, nucleotides, etc.) seem to require both genomes for complete functionality [4].

*Wolbachia* are vertically transmitted through infected oocytes [5]. Their presence in the germline allows for heritable DNA transfer from the bacteria to the metazoan host. Horizontal genetic transfers (HGTs) have been reported in several *Wolbachia-*infected filarial and arthropod species [6], [7], [8], [9], and evidence for transcription of transferred sequences has been reported in *Drosophila ananassae* that have been cured of *Wolbachia* by antibiotic treatment [7]. Similar expression studies in filarial nematodes are difficult because the infection cannot be cleared without damaging the worms. In any case, the transferred *Wolbachia* DNA sequences found in the *Wolbachia*-dependent filarial species are most likely degenerate [7] and therefore unable to produce functional proteins.

In this study, we examined two filarial nematode species from different clades [10] that are naturally *Wolbachia-*free, namely *Acanthocheilonema viteae* (a rodent parasite whose life cycle can be maintained in the laboratory) and *Onchocerca flexuosa* (a parasite of European red deer and a close relative of the agent of African river blindness) [11], [12], [13]. It has been suggested that the ancestors of these species were colonized in the distant past, as some 90% of filarial nematode species examined to date contain the bacteria [10], [11]. We hypothesized that if this is true, HGT may have brought *Wolbachia* DNA into the nuclear genomes of these species prior to endosymbiont loss. We used massively parallel sequencing to survey the genomes of *A. viteae* and *O. flexuosa* in search of *Wolbachia*-like DNA sequences. The presence of such *Wolbachia*-like sequences in their nuclear genomes provides the first direct evidence that the ancestors of these species harbored *Wolbachia* endosymbionts that were subsequently lost. Transferred *Wolbachia* genes and/or regulatory elements may help explain the ability of uninfected species to survive without a bacterial partner. Further analysis of transferred genes will provide insight into the nature of the symbiotic relationship between *Wolbachia* and its filarial nematode hosts.

## Results

### 1. Genome sequencing and identification of *Wolbachia*-like sequences

To survey the genomes of *A. viteae* and *O. flexuosa* for transferred *Wolbachia* sequences, fragment and paired-end genomic libraries were sequenced using 454 GS-FLX technology. Two orthologous approaches were undertaken to remove redundancy or capture longer contigs containing *Wolbachia* homologs (see *Materials and Methods*). *B. malayi*, the only filarial nematode for which draft genome information is available, has an ∼95 Mb genome containing ∼14% repetitive sequences [14]. Assuming a similar size and structure, we estimate that assembled contigs provide a ∼38% coverage of the non-repetitive portions of the *A. viteae* genome. It is not possible to estimate coverage of the *O. flexuosa* genome, as the coverage was insufficient for assembly of paired-end reads. BLASTN analyses identified 45 and 92 genomic fragments containing *Wolbachia*-like sequences in *A. viteae* and *O. flexuosa*, respectively (Tables 1, S1 and S2). Subsequent similarity searches found that 14 of the 45 genomic fragments in *A. viteae* and 32 of the 92 genomic fragments in *O. flexuosa* also contain filarial nematode gene homologs. This demonstrates that the *Wolbachia* homologs residing on these fragments are physically integrated into the filarial genomes.

**Table 1:** Identification of *Wolbachia*-like sequences.

Species	Library Setup	Fragments with *Wolbachia*-like sequences	*Wolbachia* Homologs	Average %ID of *Wolbachia* homologs	Average length of *Wolbachia* homologs	Fragments with Junctions*
*O. flexuosa*	Paired end	92	114	78±6%	158.9±82.6 bp	32
*A. viteae*	Fragment	45	49	81±6%	173.6±191.8 bp	14

*Fragments with junctions are defined as continuous pieces of DNA that contain sequences homologous to both *Wolbachia* and nematode genes.

### 2. Analysis of *Wolbachia-*like sequences

BLAST analysis was used to annotate the genomic DNA fragments identified in this screen (Tables S1 and S2). A total of 49 and 114 *Wolbachia-*like DNA sequences were identified in *A. viteae* and *O. flexuosa*, respectively. The average identity (% ± standard deviation) of the *Wolbachia* homologs to their top BLAST hit was 78±6% in *A. viteae* and 81±6% in *O. flexuosa*, and the average alignment length (bp ± standard deviation) was 159±83bp and 174±192 bp for *A. viteae* and *O. flexuosa*, respectively. For comparison, the average identity to a filarial nematode gene was 79±16% in *A. viteae* and 83±6% in *O. flexuosa*. Despite low-level sequence coverage, seven *Wolbachia* genes were represented by sequence fragments in both *A. viteae* and *O. flexuosa*. Some of the transferred fragments present in *A. viteae* or *O. flexuosa* also correspond to *Wolbachia* gene fragments present in the nuclear genome of *B. malayi* [7]. So far, none have been identified in all three species (Table 2).

**Table 2:** *Wolbachia* homologs found in multiple species.

Annotation	*A. viteae*		*O. flexuosa*		*B. malayi*
	DNA	RNA	DNA	RNA	DNA
rod shape-determining protein RodA	+	+	+	−	
4-Hydroxy-3-methylbut-2-enyl diphosphate reductase, IspH	+	−	+	+	
ATP-binding subunit of Clp protease and DnaK/DnaJ chaperones	+	+	+	+	
methionyl-tRNA synthetase	+	−	+	+	
ribosomal large subunit pseudouridine synthase C, putative	+	+	+	+	
ribosomal protein L27	+	+	+	n/a	
DNA-directed RNA polymerase, beta/beta' subunits	+	+	+	−	
valyl-tRNA synthetase			+	+	+
type IV secretion system protein VirB4, putative			+	+	+
ATP-dependent Zn protease, HflB			+	−	+
dimethyladenosine transferase			+	−	+
DNA polymerase III, beta subunit			+	n/a	+
cell cycle protein (ftsZ) gene			+	+	+
DNA polymerase III, gamma/tau subunit	+	+			+
IMP dehydrogenase, GuaB	+	+			+

*Wolbachia* inserts in the nuclear genome of *B. malayi* were described previously [7]. Shared homologs were identified by alignment to *Wolbachia* sequences with the same locus tag by BLASTN. Presence of sequences in the nuclear genome or among transcripts (+), lack of expression at RNA level (−), and an inability to test for expression (n/a) are noted.

### 3. Cellular processes represented by the transferred DNA

Each of the *Wolbachia*-like gene fragments identified in this study was assigned to a COG functional category in order to determine which cellular processes and pathways were most heavily represented in our transferred fragment collection (Table 3). Forty of the 49 *Wolbachia* sequences from *A. viteae* and 104 of the 114 from *O. flexuosa* could be matched to gene from the *Wolbachia* strain wBm from *B. malayi* with a functional role. No COG functional category was identified as over-represented in the HGT sequences as compared to the genome of wBm (Fisher's Exact test, Bonferroni step-down correction, p<0.01).

**Table 3:** Assignment of *Wolbachia*-like sequences to COG functional categories.

Description	*O. flexuosa*		*A. viteae*		*wBm*	
	# loci	% of total	# loci	% of total	# loci	% of total
**Information Storage and Processing**						
Translation	15	13.2%	10	20.4%	121	15.0%
Transcription	3	2.6%	2	4.1%	18	2.2%
Replication, recombination and repair	7	6.1%	6	12.2%	54	6.7%
**Cellular Processes**						
Cell cycle control, mitosis and meiosis	3	2.6%	2	4.1%	9	1.1%
Defense mechanisms	0	0.0%	0	0.0%	2	0.2%
Signal transduction mechanisms	2	1.8%	0	0.0%	10	1.2%
Cell wall/membrane biogenesis	6	5.3%	3	6.1%	33	4.1%
Cell motility	0	0.0%	0	0.0%	1	0.1%
Intracellular trafficking and secretion	7	6.1%	0	0.0%	29	3.6%
Posttranslational modification, protein turnover, chaperones	8	7.0%	1	2.0%	51	6.3%
**Metabolism**						
Energy production and conversion	10	8.8%	3	6.1%	68	8.4%
Carbohydrate transport and metabolism	1	0.9%	3	6.1%	24	3.0%
Amino acid transport and metabolism	10	8.8%	5	10.2%	38	4.7%
Nucleotide transport and metabolism	8	7.0%	2	4.1%	37	4.6%
Coenzyme transport and metabolism	2	1.8%	3	6.1%	33	4.1%
Lipid transport and metabolism	5	4.4%	2	4.1%	26	3.2%
Inorganic ion transport and metabolism	5	4.4%	0	0.0%	35	4.3%
Secondary metabolites biosynthesis, transport and catabolism	1	0.9%	0	0.0%	11	1.4%
**Poorly Characterized**						
General function prediction only	6	5.3%	3	6.1%	63	7.8%
Function unknown	5	4.4%	1	2.0%	31	3.9%
Not in COGs	8	7.0%	1	2.0%	176	21.9%
**Total**	**114**		**49**		**805**	

*Wolbachia* homologs in *A. viteae* and *O. flexuosa* were identified based on BLAST homology. The homolog was assigned to the same category as its homolog in wBm.

### 4. Potential source of transferred fragments

**Figure 1:**
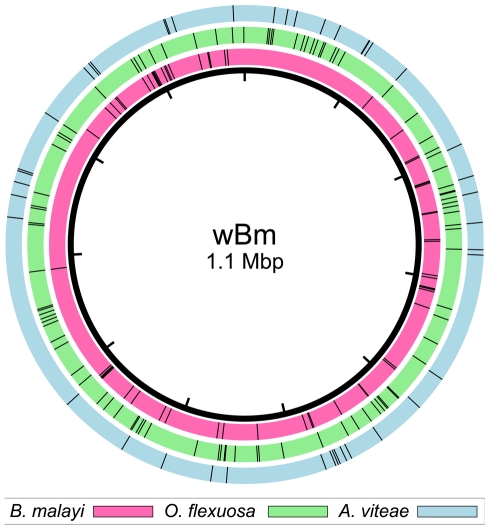
Mapping transferred fragments to a sequenced *Wolbachia* genome. Black circle represents the 1.1 Mbp genome of the *Wolbachia* endosymbiont of *B. malayi*. Tick marks in the colored outer rings indicate where a transferred DNA fragment found in the indicated species would align to the *Wolbachia* genome. Fragments found in the *B. malayi* genome were previously described by Dunning Hotopp et al. [7].

Of the 49 *Wolbachia* homologs found in *A. viteae*, 19 (39%) align best to a filarial *Wolbachia* sequence and 30 align best to an insect *Wolbachia* sequence. Likewise, 47 (41%) of the *Wolbachia* homologs in *O. flexuosa* align best to a filarial *Wolbachia* sequence and 67 align best to an insect *Wolbachia* sequence (Tables S1 and S2). Additionally, there was no apparent clustering pattern when transferred sequences were aligned to the circular genome of the *Wolbachia* endosymbiont of *B. malayi* (Fig. 1). Alignment of transferred fragments to the genomes of the *Wolbachia* endosymbionts of *Drosophila melanogaster*, *Drosophila simulans*, and *Culex pipiens* showed a similar lack of clustering. Therefore, we cannot determine whether a large piece of *Wolbachia* DNA (or even an entire bacterial genome) was inserted and subsequently fragmented and scattered in the filarial genomes over time, or if small fragments were shuttled into the genome separately.

### 5. Mechanism of transfer

**Figure 2:**
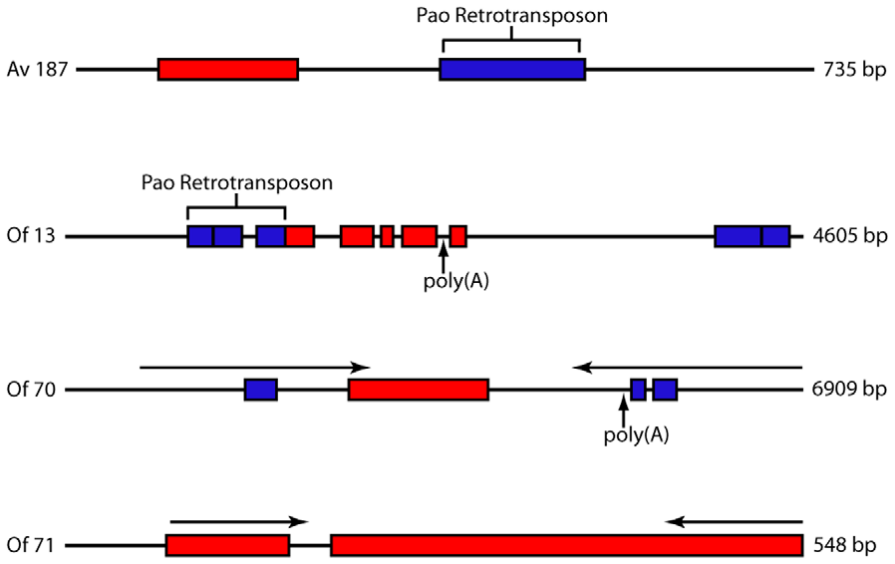
Schematic of genomic DNA fragments containing *Wolbachia* homologs. Figure outlines the structure of four genomic sequence fragments identified in this study. The exact annotation and coordinates of each of the homologs depicted can be found in Tables S1 and S2. Blue blocks represent regions of homology to nematode sequences while red blocks represent regions homologous to *Wolbachia* sequences. Horizontal arrows represent inverted repeats in the DNA sequence. Inverted repeat segments in Of70 share 88% identity with one another while the repeated segments in Of71 share 82.8% identity.

The mechanism responsible for DNA transfer from *Wolbachia* is unknown, but the sequence data provide some interesting clues (Tables S1, S2, S3, and S4). *O. flexuosa* contains several homologs to *Wolbachia* proteins involved in bacterial type IV secretion. This suggests that the DNA donor *Wolbachia* strain had a type IV secretion system which could have been capable of shuttling DNA out of the bacterial compartment. Furthermore, remnants of pao retrotransposon sequences were identified in both species (*A. viteae* contig 187 and *O. flexuosa* contig 13). For example, *O. flexuosa* contig 13 contains a *Wolbachia*-like DNA fragment flanked by a pao retrotransposon sequence and a poly(A) tract (Fig. 2). Large poly(A) sequences (>20 bases) are present in eight of our reported contigs. Most lie within a few hundred bases of a *Wolbachia*-like sequence. These poly(A) sequences may be remnants of poly(A) tails and suggest retrotransposition of processed eukaryotic mRNAs. Sequence duplication and inverted repeats present in our data are also consistent with transposon insertion sites (Fig. 2). Arthropod *Wolbachia* contain prophage sequences thought to enhance DNA rearrangements [15]; one phage sequence was detected in *A. viteae* (wAv16332).

### 6. Coding potential of transferred fragments

Most of the sequences identified in this screen represent only small portions of *Wolbachia* genes. Some of these sequences, 23 in *A. viteae* and 61 in *O. flexuosa*, are truncated at the end of a contig, so further sequencing will determine their actual length. The fragments that fall entirely within a sequenced contig (i.e. >25bp from the end of a contig) had average sizes of 146±84 and 183±219 bp in *A. viteae* and *O. flexuosa*, respectively. For comparison, the predicted protein coding genes of the *Wolbachia* endosymbiont of *B. malayi* range from 42 to 2839 amino acids; 35 proteins are predicted to be encoded by sequences shorter than 200 bp (∼66 amino acids) (www.ncbi.nlm.nih.gov/sites/entrez?Db=genome&Cmd=Retrieve&dopt=ProteinTable&list_uids=630). BLASTX was able to identify 28 *Wolbachia* homologs in *A. viteae* and 70 in *O. flexuosa*, fewer homologs than the number identified by BLASTN. Ten of the 28 in *A. viteae* and 27 of the 70 homologs in *O. flexuosa* are free of stop codons and frameshift mutations (see Tables S3 and S4).

### 7. Expression of the transferred fragments

Sybr Green qRT-PCR was used to assess expression of *Wolbachia*-like sequences regardless of the presence of an open reading frame. Stringent controls were used to rule out DNA contamination. Thirty of 42 *A. viteae* and 56 of 87 *O. flexuosa Wolbachia*-like sequences tested were expressed at the RNA level (Table S5). 14 of the expressed *Wolbachia* homologs in *A. viteae* and 34 in *O. flexuosa* appear to be transcribed from pseudogenes relative to what is known from the endobacterial genome.

### 8. Localization of transcripts

**Figure 3:**
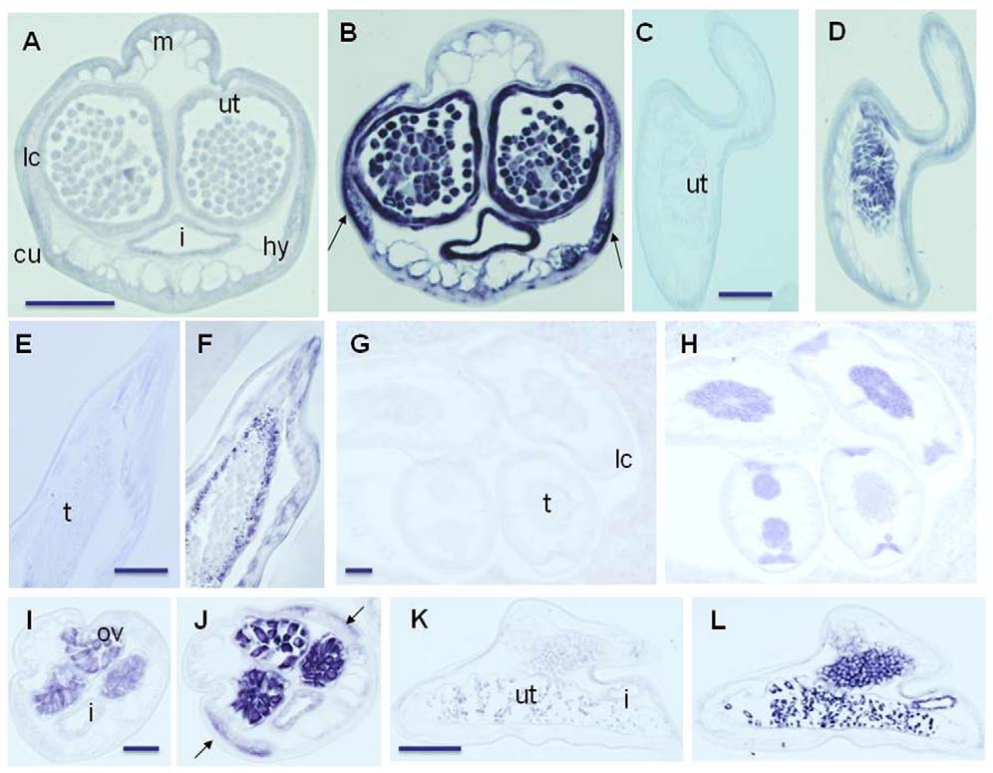
*In situ* hybridization of adult filarial worms. A–H are stained with probes made from an *O. flexuosa* sequence with homology to *Wolbachia* 2- methylthioadenine synthase (2-MAS, wOf53). A, C, E and G are stained with the sense probe (negative control), while B, D, F and H show matching consecutive sections stained with the antisense probes. 2-MAS probe labels lateral cords, intrauterine stretched microfilaria and uterine and intestinal epithelium of female *B. malayi* (B), the oocytes and uterus of female *A. viteae* (D), the spermatogonia in the periphery of the testes of male *A. viteae* (F), and lateral chords and different stages of sperm development in the testes of male *O. flexuosa* (H). I–L are stained with probes made from an *O. flexuosa* sequence with homology to *Wolbachia* DNA polymerase I (pol I, wOf88). I and K are stained with sense probes (negative control) while H and L are the matching consecutive sections stained with antisense probes. pol I probe labels ovaries and granular structures resembling *Wolbachia* (arrows) in the lateral chords of female *B. malayi* (J) and oocytes in female *A. viteae* (L). Abbreviations: m, musculature; i, intestine; lc, lateral chord; cu, cuticle; hy, hypodermis; t, testes; ut, uterus; ov, ovary. Scale bar 40 µm.

*In situ* RNA hybridization was used to localize two transcripts predicted to arise from pseudogenes of 2-methylthioadenine synthase (2-MAS), which contains a frameshift mutation, and DNA polymerase I (pol I), which contains several premature stop codons. The *Wolbachia* homologs of both of these sequences are involved in nucleic acid synthesis. The 321bp 2-MAS and 415bp polA probe sequences had 83% and 73% identity to their homologs in wBm, respectively. The 2-MAS probe labeled the lateral chords and intrauterine stretched microfilaria, tissues containing *Wolbachia* endobacteria, as well as the intestinal and uterine epithelium in *B. malayi* (Fig. 3B). The staining of the intestine and uterine epithelium could be indicative of an expressed HGT fragment in *B. malayi*, as these tissues do not contain *Wolbachia*. In *A. viteae*, this probe labeled developing embryos in females and late spermatogonia in males (Fig. 3D and 3F). No staining was seen in the lateral chords. Most of the examined *O. flexuosa* nodule sections did not contain females with developing embryos. However, one *O. flexuosa* nodule containing a young male showed intensive labeling of the testes similar to that seen in male *A. viteae* (Fig. 3F, H). The pol I probe weakly labeled *Wolbachia* in the lateral chords (arrows) of *B. malayi* (Fig. 3J). Some background staining was observed in the ovaries and uterus using the sense probe (Fig. 3 I, K), but this was very weak compared to the strong signal obtained with the anti-sense probe in female *B. malayi* and *A. viteae* (Fig. 3J, L). These results show that *Wolbachia*-like pseudogene transcripts can be detected in *Wolbachia*-free filarial species. Expression appears to be tightly regulated, because not all stages, body regions and tissue types were stained.

## Discussion

The nuclear genomes of two distantly related, *Wolbachia*-free filarial nematode species contain *Wolbachia*-like DNA sequences that were obtained from a former endosymbiont via HGT. We detected transcription of several *Wolbachia* homologs present in *A. viteae* and *O. flexuosa* despite the fact that many of these sequences are degenerate. *In situ* hybridization showed that two pseudogene transcripts had tissue-specific expression patterns in three filarial species. Our results provide strong evidence to support the hypothesis that the ancestor of extant *Wolbachia*-free filarial species was infected with *Wolbachia*.

Low coverage genome sequencing was used as a cost-effective approach to identify *Wolbachia*-like sequences. Providing draft genomes and a full inventory of all transferred DNA fragments from *A. viteae* and *O. flexuosa* was beyond the scope of this project. Rather, our aim was to provide evidence that the absence of *Wolbachia* in presently uninfected filarial species is due to secondary loss and that DNA had been passed from *Wolbachia* to these species prior to this loss. At present, draft genomes are only available for three parasitic nematode species; over 20 more are in progress [16]. Although priority is still given to pathogens of socio-economic importance, future studies may provide more complete drafts of these genomes at a later date.

The genetic screens described in this report identified 49 and 114 *Wolbachia* homologs in *A. viteae* and *O. flexuosa*, respectively. It will not be possible to determine if *O. flexuosa* and *A. viteae* have the same number of transferred genes until both genomes are fully sequenced. However, it is likely that we have identified more sequences in *O. flexuosa* due to technical issues like the use of a paired-end sequencing approach as opposed to the traditional fragment library that was used for *A. viteae*.

Several methods are commonly used to detect HGT events [17]. Approaches such as analysis of GC content or codon bias were not feasible because the GC content of filarial worms is similar to that of *Wolbachia* [4], and because we do not have enough information about the genomes of *A. viteae* and *O. flexuosa* to evaluate their codon usage. Our project relied heavily on homology-based analyses. This was ideal for our application because the suspected source of the transfer could be inferred based on the presence of *Wolbachia* in other filarial species and based on the propensity of *Wolbachia* to transfer DNA to the nuclear genomes of its hosts [7].

It is believed that mutualistic *Wolbachia* provide metabolites that are essential for host reproduction, development and survival. The combined genomes of *B. malayi* and its *Wolbachia* endosymbiont encode complete pathways for the biosynthesis of purines, pyrimidines, riboflavin, flavin adenine dinucleotide and heme, pathways that are missing or incomplete in the filarial genome alone [4]. Some of the sequence fragments we identified are homologous to genes involved in these processes (Tables S1 and S2). For instance, several genes related to heme synthesis and export were present in the HGT fragment list. Nematodes, including *C. elegans*, are not known to synthesize their own heme [18], so the presences of a complete heme biosynthetic pathway in either *A. viteae* or *O. flexuosa* would be unique. Since only portions of these genomes have been scanned, further studies are needed to determine the full insert length for many of the known *Wolbachia* homologs and to reveal more transferred sequences, some of which may encode full-length gene products.

The data provide intriguing clues about the donor strain and how transferred DNA was incorporated into the nuclear genome of filarial nematodes. If the transfer came from an extant *Wolbachia* species, the majority of the fragments would show a top BLAST score to this species. We would also expect that the transferred fragments would fall within certain portions of the donor genome if they were incorporated in one or few events. These characteristics were not seen when we compared the transferred fragments to sequenced *Wolbachia* genomes. The variability in the strain of the top BLAST hit and the average percent identity to that hit suggests that transfer(s) either came from a species that is not represented in public databases and/or that the transferred sequences have mutated over time with respect to their parent gene. Either way, these sequence changes must have taken millions of years to accumulate and stabilize in filarial and *Wolbachia* populations [19]. The initial transfer event(s) probably took place between an ancestral *Wolbachia* strain and an ancestral filarial species. It is likely that neither of these exists in the same form today, but we see the “fossilized” evidence of their interaction in the genomes of their descendents.

Questions remain as to how the DNA escaped the bacterial compartment and entered the nucleus of the host cell. It is possible that this may have involved bacterial Type IV secretion, a system conserved in *Wolbachia*. Type IV secretion systems are capable of shuttling proteins and nucleoprotein complexes across membranes and are known to facilitate gene transfer [20], [21]. After translocation, DNA may have been inserted into the host genome by transposable elements. Furthermore, the remnants of retrotransposons and large poly(A) stretches found in our sequences suggest that some of the sequences may be processed pseudogenes derived from mature mRNAs [19]. We have yet to identify the parent copies of these putative processed pseudogenes.

Many of the *Wolbachia* homologs identified in this study show insertions and/or truncations, frameshift mutations and premature stop codons relative to homologs from sequenced *Wolbachia* genomes. Gene fragmentation and degradation suggests a lack of selective pressure to maintain coding capacity, but full length transcripts and proteins are not always required for biological function. For example, transfer of DNA from mitochondria to the nuclear genome can generate novel exons that alter protein function [22]. Likewise, truncated *Wolbachia* sequences inserted into filarial genes may act as new protein domains that alter or enhance the function of existing nematode proteins. Pseudogenes containing frameshifts and premature stops are abundant in many genomes [23], and widespread transcription of pseudogenes has been reported [24], [25]. mRNA recoding mechanisms, which are employed infrequently in most organisms, could allow the translational machinery to produce a protein despite these coding errors [26]. Even if the pseudogene sequences are not translated, recent studies have shown that expressed pseudogenes sequences can regulate expression of other genes through RNA interference [27], [28], [29], [30]. Further studies will determine which of these mechanisms might allow the transferred *Wolbachia* sequences reported here to contribute to filarial biology.

We have provided strong evidence that two distantly related *Wolbachia*-free filarial nematodes contain *Wolbachia*-like DNA in their nuclear genomes. HGT from bacteria may be a relatively common phenomenon in nematode phylogeny. As previously mentioned, *Wolbachia* have inserted DNA in the nuclear genomes of endosymbiont-dependent filarial nematode hosts [7]. In these cases, the source of the transfer is obvious because the mutualistic relationship between the two organisms has been maintained, but the functions of the inserted sequences are still unknown. The converse situation exists in plant parasitic nematodes. Cellulase enzymes that support plant parasitism were probably obtained from bacteria via HGT [31], [32]. These cellulase genes show a high degree of homology to genes from a wide range of bacterial species. The exact source of the transferred DNA is difficult to pinpoint because the relationship between the DNA donor and recipient has not been maintained. The case of *A. viteae* and *O. flexuosa* is special because the relationship with the DNA donor has not been maintained, but the donor can be easily identified by BLAST homology and by the presence of *Wolbachia* in most filarial species. Future studies will generate a comprehensive list of transferred fragments in several *Wolbachia-*free filarial species. This information may identify key genes and pathways that explain the fascinating and medically important symbiotic relationship between filarial worms and *Wolbachia* endobacteria.

## Materials and Methods

### 1. Parasite material and DNA isolation

Adult *O. flexuosa* worms were collected from red deer (*Cervus elaphus*) in Germany (Schleswig-Holstein). Adult worms were dissected from nodules collected from freshly shot deer [13]. Adult *A. viteae* and *B. malayi* were obtained from experimentally infected Mongolian gerbils as previously described [33], [34]. DNA was isolated from adult worms using the DNeasy Blood and Tissue Kit (Qiagen, Valencia, CA).

### 2. Library construction, sequencing and BLAST analysis

A fragment library was created from 15 µg of *A. viteae* DNA. Two runs on a Genome Sequencer FLX (454 Life Sciences/Roche Diagnostics, Branford, CT) using standard FLX chemistry generated 768,909 reads and a total of 181 Mbp of sequence. Sff files were deposited in the NCBI short read archive (SRX001994). Newbler 1.1.03.24 assembled reads into 68,805 contigs containing ∼31 Mbp of sequence using default parameters. The largest contig was 6.7 kbp and the average size was 0.9 kbp. Contigs were analyzed by BLASTN against the Genbank nucleotide collection (nt) and by BLASTX against the Genbank non-redundant protein database (nr) using NCBI BLASTALL to identify regions with a top hit to *Wolbachia* with an e-value less than 1×10^−5^. This cutoff was used for all BLAST searches. All *A. viteae* contigs larger than 600 bp were also split into smaller fragments (optimal maximum and minimum were set to 600 and 300 bp, respectively) using a custom Perl script and re-analyzed by BLASTN (nt_05292009) and BLASTX (nr_05292009) to ensure that all *Wolbachia* homologs were identified despite strong homology to different genes/proteins in other regions of the sequence. Contigs of interest were PCR amplified and cloned using the TOPO TA Cloning Kit for Sequencing or the TOPO-XL PCR Cloning Kit (Invitrogen, Carlsbad, CA, USA), and sequenced for verification. Sequences are posted on the Whole Genome Shotgun FTP site on Nematode.net (www.nematode.net/FTP/index.php).

*O. flexuosa* paired-end genomic DNA libraries were constructed as previously described [35] using 5.0 µg of DNA. One run on a Genome Sequencer FLX using standard FLX chemistry produced 516,745 reads containing 135 Mbp of sequence including adapter sequences. Sff files were deposited in the NCBI short read archive (SRX015550). A custom Perl script was designed for removal of the 44 bp adapter and separation of the paired end (PE) sequences which were then analyzed by BLASTX (nr_05292009) and BLASTN (nt_05292009). Complete, unsorted reads were also analyzed by BLASTX (nr_09182007) and BLASTN (nt_04062009) using WU-BLAST 2.0. 137 reads containing *Wolbachia*-like sequences were identified. Read sequences were used to amplify larger portions of the *O. flexuosa* genome. After sequencing the larger genomic fragments, redundant/overlapping fragments were collapsed using Contig Express (Invitrogen). This produced 42 large fragments (those amplified using primers matching to PE sequences) ranging in size from 1124 to 7725 bp with an average size of 3028±1386 bp and 50 smaller sequences (those representing assembled or unassembled portions of a PE read) ranging in size from 55 to 548 bp with an average size of 243±83 bp. All fragments sequences are posted on the Data FTP page of Nematode.net.

### 3. Assessment of COG functional roles and mapping to the genome of the *Wolbachia* endosymbiont of *B. malayi*

*Wolbachia*-like sequences were compared against the genome of the *Wolbachia* endosymbiont strain TRS of *B*. *malayi* (wBm) using BLASTN, and the locus tag of the top hit was recorded. The *Wolbachia*-like sequence was assigned to the same COG functional category as its wBm homolog as reported in NCBI Entrez. The Open Source Python (http://www.python.org/) library ReportLab (http://www.reportlab.com/software/opensource/rl-toolkit/) was used to generate a figure that marks the position of the homologous locus. Tick marks represent the midpoint of the coordinates for each locus tag as extracted from the GenBank data file for the wBm genome (NC_006833).

### 4. RNA isolation, cDNA synthesis and qRT-PCR

Adult worms were homogenized in 1 mL Trizol (Invitrogen), and RNA was isolated using organic extraction followed by column purification using an RNeasy Mini Kit (Qiagen) including the on-column DNase digest. A second DNase treatment was performed using Ambion's DNA-*free* DNase kit (Applied Biosystems, Austin, TX, USA). rRNA was depleted using the RiboMinus Eukaryote Kit for RNA-Seq (Invitrogen). Samples were tested for DNA contamination by conventional PCR using 35 cycles to ensure that no products were amplified. cDNA was made from 500 ng mRNA using qScript cDNA super mix (Quanta Biosciences, Gaithersburg, MD, USA). Conventional PCR assays using primers designed to span introns were used to test for DNA contamination again. Intron-spanning primer sequences are provided in Table S6. These target sequences from *O. flexuosa* with homology to hypothetical proteins in *B. malayi* that were readily amplifiable from *O. flexuosa* genomic and cDNA. Only Of_IC 1 worked well with *A. viteae* mRNA, which was also rigorously tested with wAv primers used in this study prior to cDNA synthesis (see Table S6). Specific *Wolbachia* sequences were detected in cDNA by SybrGreen qRT-PCR using PerfeC_T_a SYBR Green FastMix, ROX (Quanta Biosciences). Primer sequences are reported in Table S6. Reactions were done in duplicate to ensure accuracy, and all qRT-PCR experiments included a 10 ng DNA positive control, 10−0.1 ng ten-fold dilutions of cDNA, and a 10 ng RNA negative control. Dissociation curves were examined to rule out non-specific amplification, and all products were assessed by agarose gel electrophoresis. Sequences were reported as expressed at the transcript level when signal for the 0.1 ng cDNA template crossed the cycle threshold at least 3 cycles before the mRNA negative control.

### *5. In situ* hybridization

Adult *B. malayi* and *A. viteae* worms and *O. flexuosa* nodules were fixed for 24–72 h in DEPC-treated 4% buffered formaldehyde and embedded in paraffin using standard histological procedures. Two sequences were amplified from *O. flexuosa* cDNA and cloned into a dual promoter PCRII plasmid (Invitrogen). Primer sequences are reported in Table S6. After linearization of the plasmid, biotinylated antisense probes and sense negative controls were prepared with Ambion Megascript T7 and Sp6 high yield transcription kits (Applied Biosystems). Following DNase digestion (Roche, Indianapolis, IN, USA), probes were concentrated by ethanol precipitation, re-suspended in DEPC-treated water, and stored at −20°C. Sections (5 µm) were deparaffinized, digested with pepsin HCl for 7 min, and hybridized at 37°C overnight in a humid chamber with 1 µg of RNA probe in hybridization buffer (50% formamide, 5XSSC, 0.3 mg/ml yeast tRNA, 100 µg/ml heparin, 1× Denhardts Solution, 0.1% Tween 20, 0.1% CHAPS and 5 mM EDTA). A stringency wash was performed at 60°C for 30 min, and detection was performed using the ‘In situ Hybridization Detection System’ (K0601, DakoCytomation, Hamburg, Germany). Sections were incubated for 20 min with streptavidin-AP conjugate at room temperature. BCIP/NBT substrate solution was used for 10–30 min to visualize the RNA target. Sections were examined using an Olympus-BX40 microscope (Olympus, Tokyo, Japan) and photographed with an Olympus DP70 microscope digital camera.

## Supporting Information

Table S1 BLASTN annotation of Acanthocheilonema viteae genomic DNA fragments. BLASTN based annotation of all A. viteae contigs that contain Wolbachia homologs with an e-value less than 1e-05. Annotation given is that of the top blast hit unless description of top hit was uninformative. In this case, the annotation of a subsequent hit from the same region was taken instead. Abbreviations are as follows: Wolbachia endosymbiont of Culex quinquefasciatus, wCq; Wolbachia endosymbiont of Brugia malayi, wBm; Wolbachia endosymbiont of Drosophila simulans, wRi; Wolbachia endosymbiont of Drosophila melanogaster, wDm; Wolbachia endosymbiont of Onchocerca volvulus, wOv; Wolbachia endosymbiont of Dirofilaria immitis, wDi. The average length of a sequence with homology to a Wolbachia gene was 158.9 plus or minus 82.6bp. The average percent identity of an A. viteae sequence to a Wolbachia gene was 78.0 plus or minus 6.0%, while the average percent identity to a nematode gene was 85.2 plus or minus 4.5%. The difference between the average size of a Wolbachia homolog and a nematode homolog was statistically significant according to Student's t-test (p-value = 1.42 e-07).
(0.11 MB DOC)

Table S2 BLASTN annotation Onchocerca flexuosa genomic DNA fragments. BLASTN based annotation of all O. flexuosa contigs and read sequences that contain Wolbachia homologs with an e-value less than 1e-05. Annotation given is that of the top blast hit unless description of top hit was uninformative. In this case, the annotation of a subsequent hit from the same region was taken instead. Abbreviations are as follows: Wolbachia endosymbiont of Drosophila simulans, wRi; Wolbachia endosymbiont of Brugia malayi, wBm; Wolbachia endosymbiont of Onchocerca volvulus, wOv; Wolbachia endosymbiont of Culex quinquefasciatus, wCq; Wolbachia endosymbiont of Dirofilaria immitis, wDi; Wolbachia endosymbiont of Drosophila melanogaster, wDm. The average length of a sequence with homology to a Wolbachia gene was 173.6 plus or minus 191.8bp. The average percent identity of an O.flexuosa sequence to a Wolbachia gene was 80.6 plus or minus 6.0%, while the average percent identity to a nematode gene was 83.1 plus or minus 6.1%. This difference was statistically significant according to Student's t-test (p-value = .0014).
(0.15 MB DOC)

Table S3 BLASTX annotation of Acanthocheilonema viteae genomic DNA fragments. BLASTX based annotation of all A. viteae contigs containing Wolbachia homologs with a BLASTN e-value less than 1e-05. All hits to Wolbachia genes by BLASTX were recorded, regardless of e-value. Abbreviations are as follows: Wolbachia endosymbiont of Culex quinquefasciatus, wCq; Wolbachia endosymbiont of Drosophila simulans, wRi; Wolbachia endosymbiont of Brugia malayi, wBm; Wolbachia endosymbiont of Drosophila willistoni, wDw; Wolbachia endosymbiont of Muscidifurax uniraptor, wMu; Wolbachia endosymbiont of Onchocerca volvulus, wOv; Wolbachia endosymbiont of Armadillium vulgare, wAv. The average length of a sequence with homology to a Wolbachia protein was 123.8 plus or minus 73.5bp. The average percent ID to a Wolbachia protein was 62.3 plus or minus 13.8%. According to Student's t-test, this is significantly lower than the average percent identity to a nematode protein, 79.1 plus or minus 15.6% (p-value = .0001). The Student's t-test indicates that the average percent identity to a Wolbachia protein is also significantly lower than the percent identity of a sequence to a Wolbachia gene on the nucleotide level (p-value = 8e-10).
(0.13 MB DOC)

Table S4 BLASTX annotation of O. flexuosa genomic DNA fragments. BLASTX based annotation of all O. flexuosa genomic fragmetns containing Wolbachia homologs with a BLASTN e-value better than 1e-05. All hits to Wolbachia genes by BLASTX were recorded, regardless of e-value. Abbreviations are as follows: Wolbachia endosymbiont of Drosophila simulans, wRi; Wolbachia endosymbiont of Onchocerca volvulus, wOv; Wolbachia endosymbiont of Drosophila melanogaster, wDm; Wolbachia endosymbiont of Brugia malayi, wBm; Wolbachia endosymbiont of Culex quinquefasciatus, wCq; Wolbachia endosymbiont of Dirofilaria immitis, wDi; Wolbachia endosymbiont of Drosophila willistoni, wDw; Wolbachia endosymbiont of Muscidifurax uniraptor, wMu. The average length of a sequence with homology to a Wolbachia protein was 148.3 plus or minus 121.2bp. The average percent ID to a Wolbachia protein was 67.3 plus or minus 13.2%. According to Student's t-test, this is significantly lower than the average percent identity to a nematode protein, 76.3 plus or minus 16.0% (p-value = .00014). The Student's t-test indicates that the average percent identity to a Wolbachia protein is also significantly lower than the percent identity of a sequence to a Wolbachia gene on the nucleotide level (p-value = 2.03e-15).
(0.15 MB DOC)

Table S5 Results of qPCR expression studies and presence of potential open reading frames. Table describes the results of qRT-PCR studies used to determine whether Wolbachia homologs described in Tables S1 and S2 are expressed at the transcript level. + indicates that the sequenced is expressed at the RNA level while - indicates that it was not. n/a indicates that the sequence could not be tested using this method (for example, the sequence was too short or AT rich to design qRT-PCR primers). Sequences more than 25bp from the end of a genomic fragment are considered internal and sequences lacking premature stop codons and frameshift mutations are reported as potential open reading frames. Defined start and stop codons were not required for classification as a potential open reading frame.
(0.15 MB DOC)

Table S6 SYBR green qRT-PCR and RNA in situ hybridization probe primers.
(0.14 MB DOC)
