# Supplementary material for: Endosymbiont DNA in Endobacteria-Free Filarial Nematodes Indicates Ancient Horizontal Genetic Transfer
Source: PLoS One. 2010 Jun 9;5(6):e11029. doi: 10.1371/journal.pone.0011029 (PMC2882956; doi:10.1371/journal.pone.0011029)
Supplement: Table S1 — BLASTN annotation of Acanthocheilonema viteae genomic DNA fragments. BLASTN based annotation of all A. viteae contigs that contain Wolbachia homologs with an e-value less than 1e-05. Annotation given is that of the top blast hit unless description of top hit was uninformative. In this case, the annotation of a subsequent hit from the same region was taken instead. Abbreviations are as follows: Wolbachia endosymbiont of Culex quinquefasciatus, wCq; Wolbachia endosymbiont of Brugia malayi, wBm; Wolbachia endosymbiont of Drosophila simulans, wRi; Wolbachia endosymbiont of Drosophila melanogaster, wDm; Wolbachia endosymbiont of Onchocerca volvulus, wOv; Wolbachia endosymbiont of Dirofilaria immitis, wDi. The average length of a sequence with homology to a Wolbachia gene was 158.9 plus or minus 82.6bp. The average percent identity of an A. viteae sequence to a Wolbachia gene was 78.0 plus or minus 6.0%, while the average percent identity to a nematode gene was 85.2 plus or minus 4.5%. The difference between the average size of a Wolbachia homolog and a nematode homolog was statistically significant according to Student's t-test (p-value = 1.42 e-07). (0.11 MB DOC) [file pone.0011029.s001.doc]

**Table S1.** BLASTN annotation of *Acanthocheilonema viteae* genomic DNA fragments

| **Contig #** | **Length (bp)** | **Locus Name** | **Best Annotation** | **Homolog Species** | **5' coord.** | **3' coord.** | **e value** | **%ID** |
| --- | --- | --- | --- | --- | --- | --- | --- | --- |
| 187 | 739 | wAv187 | [disulfide bond formation protein, DsbB family](http://www.ncbi.nlm.nih.gov/entrez/viewer.fcgi?val=190356750&db=Nucleotide&from=961374&to=961865&view=gbwithparts&RID=7P83EXPZ012) | *wCq* | 79 | 216 | 2.0E-09 | 70% |
|  |  | Av187 | Pao retrotransposon peptidase family protein | *B. malayi* | 353 | 496 | 2.0E-09 | 71% |
| 2452 | 1054 | wAv2452a | [ribosomal protein L27](http://www.ncbi.nlm.nih.gov/entrez/viewer.fcgi?val=58418577&db=Nucleotide&from=910282&to=910539&view=gbwithparts&RID=7P83EXPZ012) | *wBm* | 18 | 147 | 1.0E-29 | 83% |
|  |  | wAv2452b | [hypothetical protein, IS5 family transposase](http://www.ncbi.nlm.nih.gov/entrez/viewer.fcgi?val=225591853&db=Nucleotide&from=914190&to=914450&view=gbwithparts&RID=9DVBUHSZ014) | *wRi* | 285 | 354 | 5.0E-05 | 78% |
| 3046 | 978 | wAv3046 | [isoleucyl-trna synthetase](http://www.ncbi.nlm.nih.gov/entrez/viewer.fcgi?val=190356750&db=Nucleotide&from=681762&to=684932&view=gbwithparts&RID=7P83EXPZ012) | *wCq* | 409 | 541 | 8.0E-26 | 82% |
| 4552 | 455 | wAv4552.1 | [predicted Zn-dependent protease, TldD ortholog](http://www.ncbi.nlm.nih.gov/entrez/viewer.fcgi?val=58418577&db=Nucleotide&from=952385&to=953812&view=gbwithparts&RID=7P83EXPZ012) | *wRi* | 3 | 180 | 1.0E-16 | 71% |
|  |  | wAv4552.2 |  |  | 235 | 300 | 7.0E-05 | 78% |
|  |  | wAv4552.3 |  |  | 303 | 453 | 4.0E-23 | 77% |
| 4766 | 903 | Av4766 | hypothetical protein | *B. malayi* | 245 | 320 | 9.0E-13 | 84% |
|  |  | wAv4766 | [hypothetical protein](http://www.ncbi.nlm.nih.gov/entrez/viewer.fcgi?val=190356750&db=Nucleotide&from=843145&to=844365&view=gbwithparts&RID=7P83EXPZ012) | *wCq* | 538 | 646 | 3.0E-19 | 81% |
|  |  | Av4766 | hypothetical protein | *B. malayi* | 722 | 903 | 5.0E-54 | 86% |
| 5106 | 2297 | wAv5106 | [transketolase, ribosomal protein S4](http://www.ncbi.nlm.nih.gov/entrez/viewer.fcgi?val=42410857&db=Nucleotide&from=369209&to=371281&view=gbwithparts&RID=7P83EXPZ012) | *wDm* | 1 | 216 | 3.0E-38 | 77% |
|  |  | Av5106 | hypothetical protein | *B. malayi* | 875 | 928 | 2.0E-07 | 87% |
| 9153 | 1261 | Av9153.1 | hypothetical protein | *B. malayi* | 1 | 167 | 1.0E-41 | 83% |
|  |  | Av9153.2 |  |  | 335 | 417 | 1.0E-17 | 86% |
|  |  | Av9153.3 |  |  | 707 | 939 | 5.0E-79 | 89% |
|  |  | wAv9153 | [DNA polymerase III, gamma/tau subunit](http://www.ncbi.nlm.nih.gov/entrez/viewer.fcgi?val=58418577&db=Nucleotide&from=563959&to=565443&view=gbwithparts&RID=7P83EXPZ012) | *wOv* | 954 | 1260 | 3.0E-63 | 79% |
| 9497 | 785 | wAv9497 | [penicillin-binding protein](http://www.ncbi.nlm.nih.gov/entrez/viewer.fcgi?val=190356750&db=Nucleotide&from=619039&to=620595&view=gbwithparts&RID=7P83EXPZ012) | *wCq* | 2 | 197 | 9.0E-38 | 78% |
|  |  | Av9497.1 | Protein kinase domain containing protein | *B. malayi* | 267 | 437 | 3.0E-57 | 90% |
|  |  | Av9497.2 |  |  | 543 | 764 | 8.0E-58 | 83% |
| 9524 | 933 | wAv9524a | [Methionyl-tRNA synthetase](http://www.ncbi.nlm.nih.gov/entrez/viewer.fcgi?val=225591853&db=Nucleotide&from=467460&to=469106&view=gbwithparts&RID=7P83EXPZ012) | *wRi* | 317 | 473 | 2.0E-27 | 78% |
|  |  | wAv9524b | [Phenylalanyl-tRNA synthetase beta chain](http://www.ncbi.nlm.nih.gov/entrez/viewer.fcgi?val=225591853&db=Nucleotide&from=341543&to=343882&view=gbwithparts&RID=7P83EXPZ012) | *wRi* | 697 | 867 | 4.0E-10 | 68% |
| 10096 | 1382 | wAv10096.1 | [ATP-dependent exoDNAse (exonuclease V) beta subunit, RecB](http://www.ncbi.nlm.nih.gov/entrez/viewer.fcgi?val=58418577&db=Nucleotide&from=218134&to=221493&view=gbwithparts&RID=7P83EXPZ012) | *wBm* | 524 | 987 | 9.0E-51 | 67% |
|  |  | wAv10096.2 |  |  | 990 | 1092 | 3.0E-06 | 75% |
| 10682 | 241 | wAv10682 | [4-hydroxy-3-methylbut-2-enyl diphosphate reductase](http://www.ncbi.nlm.nih.gov/entrez/viewer.fcgi?val=225591853&db=Nucleotide&from=1336345&to=1337274&view=gbwithparts&RID=7P9KU2FA01S) | *wRi* | 37 | 153 | 1.0E-18 | 79% |
| 11045 | 1235 | wAv11045 | [NADH dehydrogenase I, K subunit](http://www.ncbi.nlm.nih.gov/entrez/viewer.fcgi?val=190356750&db=Nucleotide&from=1061415&to=1061723&view=gbwithparts&RID=7P9KU2FA01S) | *wCq* | 285 | 400 | 4.0E-24 | 82% |
|  |  | Av11045.1 | MGC80088 protein | *B. malayi* | 424 | 639 | 2.0E-60 | 84% |
|  |  | Av11045.2 |  |  | 741 | 869 | 3.0E-31 | 84% |
|  |  | Av11045.3 |  |  | 1075 | 1235 | 8.0E-58 | 91% |
| 11910 | 483 | wAv11910 | [Actin-like ATPase involved in cell morphogenesis, MreB](http://www.ncbi.nlm.nih.gov/entrez/viewer.fcgi?val=58418577&db=Nucleotide&from=194956&to=196032&view=gbwithparts&RID=7P9KU2FA01S) | *wBm* | 43 | 468 | 2.0E-60 | 70% |
| 13336 | 1185 | Av13336 | DOMON domain containing protein | *B. malayi* | 1 | 55 | 9.0E-13 | 92% |
|  |  |  |  |  | 143 | 352 | 1.0E-62 | 86% |
|  |  | wAv13336a | [uncharacterized protein conserved in bacteria](http://www.ncbi.nlm.nih.gov/entrez/viewer.fcgi?val=58418577&db=Nucleotide&from=94156&to=94413&view=gbwithparts&RID=7P9KU2FA01S) | *wBm* | 469 | 604 | 1.0E-18 | 77% |
|  |  | wAv13336b | [3-polyprenyl-4-hydroxybenzoate decarboxylase](http://www.ncbi.nlm.nih.gov/entrez/viewer.fcgi?val=58418577&db=Nucleotide&from=94414&to=94977&view=gbwithparts&RID=7P9KU2FA01S) | *wBm* | 647 | 745 | 2.0E-26 | 89% |
|  |  | Av13336 | DOMON domain containing protein | *B. malayi* | 931 | 1101 | 2.0E-41 | 83% |
| 16332 | 607 | wAv16332 | [phage uncharacterized protein](http://www.ncbi.nlm.nih.gov/entrez/viewer.fcgi?val=190356750&db=Nucleotide&from=1079829&to=1081262&view=gbwithparts&RID=7P9KU2FA01S) | *wCq* | 179 | 491 | 2.0E-45 | 73% |
| 16679 | 1339 | wAv16679 | partial genomic sequence clone WDi318 | *wDi* | 633 | 831 | 5.0E-48 | 84% |
| 16952 | 890 | wAv16952 | [DNA-directed RNA polymerase, beta/beta' subunits](http://www.ncbi.nlm.nih.gov/entrez/viewer.fcgi?val=190356750&db=Nucleotide&from=594197&to=602716&view=gbwithparts&RID=7P9KU2FA01S) | *wCq* | 7 | 169 | 9.0E-32 | 79% |
| 19080 | 1434 | Av19080.1 | SH2 domain containing protein | *B. malayi* | 130 | 268 | 6.0E-34 | 84% |
|  |  | Av19080.2 |  |  | 510 | 643 | 2.0E-40 | 88% |
|  |  | Av19080.3 |  |  | 1203 | 1278 | 1.0E-10 | 83% |
|  |  | wAv19080 | [2-oxoglutarate dehydrogenase E1 component](http://www.ncbi.nlm.nih.gov/entrez/viewer.fcgi?val=225591853&db=Nucleotide&from=1422279&to=1424924&view=gbwithparts&RID=7P9KU2FA01S) | *wRi* | 1289 | 1390 | 3.0E-19 | 84% |
| 20032 | 669 | wAv20032 | [lipoyl synthase](http://www.ncbi.nlm.nih.gov/entrez/viewer.fcgi?val=225591853&db=Nucleotide&from=426208&to=427071&view=gbwithparts&RID=7P9KU2FA01S) | *wRi* | 7 | 137 | 1.0E-18 | 77% |
| 21852 | 241 | wAv21852 | [transketolase](http://www.ncbi.nlm.nih.gov/entrez/viewer.fcgi?val=58418577&db=Nucleotide&from=864536&to=866500&view=gbwithparts&RID=7P9KU2FA01S) | *wBm* | 51 | 255 | 2.0E-27 | 76% |
| 22549 | 242 | wAv22594 | [rod shape-determining protein, RodA](http://www.ncbi.nlm.nih.gov/entrez/viewer.fcgi?val=190356750&db=Nucleotide&from=1083736&to=1084839&view=gbwithparts&RID=7PB31WAM012) | *wCq* | 20 | 129 | 1.0E-17 | 79% |
| 22942 | 608 | wAv22942 | [pyruvate phosphate dikinase](http://www.ncbi.nlm.nih.gov/entrez/viewer.fcgi?val=190356750&db=Nucleotide&from=1274446&to=1277073&view=gbwithparts&RID=7PB31WAM012) | *wCq* | 3 | 62 | 4.0E-11 | 88% |
|  |  | Av22942 | molting L3 larva cDNA | *O. volvulus* | 184 | 262 | 2.0E-19 | 88% |
| 24238 | 691 | wAv24238 | [NADH dehydrogenase I, G subunit](http://www.ncbi.nlm.nih.gov/entrez/viewer.fcgi?val=190356750&db=Nucleotide&from=1167401&to=1169449&view=gbwithparts&RID=7PCT55K001N) | *wCq* | 102 | 284 | 4.0E-24 | 73% |
|  |  | Av24238 | RNA polymerase II subunit | *B. malayi* | 298 | 482 | 3.0E-56 | 87% |
| 24716 | 1676 | wAv24716 | partial genomic sequence, clone WDi239 | *wDi* | 672 | 797 | 1.0E-30 | 84% |
|  |  | Av24716.1 | transcription factor Ash2 | *B. malayi* | 1243 | 1329 | 3.0E-19 | 86% |
|  |  | Av24716.2 |  |  | 1442 | 1549 | 1.0E-30 | 88% |
| 25554 | 240 | wAv25554 | [carbamoyl-phosphate synthase, small subunit](http://www.ncbi.nlm.nih.gov/entrez/viewer.fcgi?val=42410857&db=Nucleotide&from=659511&to=660710&view=gbwithparts&RID=7PCT55K001N) | *wDm* | 17 | 205 | 6.0E-15 | 69% |
| 28511 | 796 | wAv28511 | [cysteine desulfurase](http://www.ncbi.nlm.nih.gov/entrez/viewer.fcgi?val=190356750&db=Nucleotide&from=1082446&to=1083687&view=gbwithparts&RID=7PCT55K001N) | *wCq* | 9 | 130 | 4.0E-17 | 78% |
| 29055 | 465 | wAv29055 | [undecaprenyl diphosphate synthase](http://www.ncbi.nlm.nih.gov/entrez/viewer.fcgi?val=225591853&db=Nucleotide&from=367785&to=368480&view=gbwithparts&RID=7PCT55K001N) | *wRi* | 7 | 220 | 7.0E-52 | 82% |
|  |  | Av29055 | electron transfer flavoprotein-ubiquinone oxidoreductase | *B. malayi* | 354 | 465 | 7.0E-33 | 89% |
| 30005 | 982 | wAv30005 | [Nuclease subunit of the excinuclease complex, UvrC](http://www.ncbi.nlm.nih.gov/entrez/viewer.fcgi?val=58418577&db=Nucleotide&from=471825&to=473645&view=gbwithparts&RID=7PCT55K001N) | *wOv* | 183 | 325 | 4.0E-23 | 77% |
| 30574 | 241 | wAv30574a | [Predicted protein](http://www.ncbi.nlm.nih.gov/entrez/viewer.fcgi?val=58418577&db=Nucleotide&from=632376&to=633641&view=gbwithparts&RID=7PCT55K001N) | *wBm* | 69 | 128 | 1.0E-10 | 88% |
|  |  | wAv30574b | Exonuclease III, predicted permease | *wBm* | 127 | 209 | 3.0E-13 | 84% |
| 31107 | 585 | wAv31107 | [type II secretion system protein, putative](http://www.ncbi.nlm.nih.gov/entrez/viewer.fcgi?val=225591853&db=Nucleotide&from=255500&to=257002&view=gbwithparts&RID=7PCT55K001N) | *wRi* | 44 | 166 | 7.0E-27 | 84% |
|  |  | Av31107 | FRG1 protein homolog | *B. malayi* | 442 | 533 | 1.0E-17 | 83% |
| 31515 | 225 | wAv31515 | [Pseudouridylate synthases, 23S RNA-specific](http://www.ncbi.nlm.nih.gov/entrez/viewer.fcgi?val=58418577&db=Nucleotide&from=273242&to=274411&view=gbwithparts&RID=7PCT55K001N) | *wBm* | 18 | 144 | 1.0E-24 | 81% |
| 31988 | 174 | wAv31988 | [ubiquinol-cytochrome c reductase, cytochrome c1](http://www.ncbi.nlm.nih.gov/entrez/viewer.fcgi?val=225591853&db=Nucleotide&from=1195555&to=1196313&view=gbwithparts&RID=7PDUG3B801S) | *wRi* | 16 | 174 | 6.0E-26 | 78% |
| 35539 | 233 | wAv35539 | [hypothetical protein](http://www.ncbi.nlm.nih.gov/entrez/viewer.fcgi?val=225591853&db=Nucleotide&from=70915&to=71325&view=gbwithparts&RID=7PDUG3B801S) | *wRi* | 96 | 223 | 4.0E-21 | 79% |
| 36441 | 380 | wAv36441 | [Na+/alanine symporter](http://www.ncbi.nlm.nih.gov/entrez/viewer.fcgi?val=58418577&db=Nucleotide&from=552576&to=553952&view=gbwithparts&RID=7PDUG3B801S) | *wBm* | 1 | 115 | 6.0E-13 | 75% |
| 38375 | 1406 | wAv38375 | [endonuclease III](http://www.ncbi.nlm.nih.gov/entrez/viewer.fcgi?val=225591853&db=Nucleotide&from=681124&to=681762&view=gbwithparts&RID=7PDUG3B801S) | *wRi* | 540 | 596 | 1.0E-09 | 87% |
| 38543 | 467 | wAv38543 | [modification methylase, HemK family](http://www.ncbi.nlm.nih.gov/entrez/viewer.fcgi?val=225591853&db=Nucleotide&from=11460&to=12314&view=gbwithparts&RID=7PDUG3B801S) | *wRi* | 33 | 256 | 6.0E-26 | 71% |
| 41791 | 217 | wAv41791 | [NADPH-dependent glutamate synthase beta chain](http://www.ncbi.nlm.nih.gov/entrez/viewer.fcgi?val=58418577&db=Nucleotide&from=69301&to=72618&view=gbwithparts&RID=7PDUG3B801S) | *wBm* | 83 | 211 | 2.0E-13 | 73% |
| 42190 | 336 | wAv42190 | [IMP dehydrogenase, GuaB](http://www.ncbi.nlm.nih.gov/entrez/viewer.fcgi?val=58418577&db=Nucleotide&from=683292&to=684788&view=gbwithparts&RID=7PDUG3B801S) | *wBm* | 13 | 190 | 1.0E-21 | 74% |
| 46345 | 259 | wAv46345 | [ubiquinol-cytochrome c reductase, cytochrome b](http://www.ncbi.nlm.nih.gov/entrez/viewer.fcgi?val=190356750&db=Nucleotide&from=800666&to=801892&view=gbwithparts&RID=7PDUG3B801S) | *wCq* | 2 | 248 | 7.0E-50 | 78% |
| 48068 | 229 | wAv48068 | [Glycyl-tRNA synthetase, alpha subunit, beta subunit](http://www.ncbi.nlm.nih.gov/entrez/viewer.fcgi?val=58418577&db=Nucleotide&from=468889&to=469728&view=gbwithparts&RID=7PDUG3B801S) | *wBm* | 124 | 228 | 3.0E-16 | 78% |
| 52396 | 242 | wAv52396 | [trna pseudouridine synthase b](http://www.ncbi.nlm.nih.gov/entrez/viewer.fcgi?val=190356750&db=Nucleotide&from=905918&to=906979&view=gbwithparts&RID=7PDUG3B801S) | *wCq* | 94 | 207 | 8.0E-18 | 79% |
| 55693 | 473 | wAv55693 | [ATP-dependent Clp protease, ATP-binding subunit ClpB](http://www.ncbi.nlm.nih.gov/entrez/viewer.fcgi?val=225591853&db=Nucleotide&from=216135&to=218735&view=gbwithparts&RID=7PDUG3B801S) | *wRi* | 170 | 314 | 7.0E-25 | 78% |
| 62916 | 317 | wAv62916 | [Porphobilinogen deaminase](http://www.ncbi.nlm.nih.gov/entrez/viewer.fcgi?val=58418577&db=Nucleotide&from=1026192&to=1027070&view=gbwithparts&RID=7PDUG3B801S) | *wBm* | 1 | 217 | 1.0E-40 | 78% |
| 64666 | 1482 | Av64666 | MIZ zinc finger family protein | *B. malayi* | 328 | 432 | 7.0E-08 | 75% |
|  |  | wAv64666 | Onchocerca Wolbachia Sequence Fragment OW4 | *wOv* | 1058 | 1151 | 2.0E-19 | 85% |
| 67545 | 102 | wAv67545 | [ATP-dependent DNA helicase RecG](http://www.ncbi.nlm.nih.gov/entrez/viewer.fcgi?val=190356750&db=Nucleotide&from=518987&to=521008&view=gbwithparts&RID=7PMCRVWD013) | *wCq* | 767 | 1062 | 9.0E-16 | 66% |

BLASTN based annotation of all *A. viteae* contigs that contain *Wolbachia* homologs with an e-value less than 1e-05. Annotation given is that of the top blast hit unless description of top hit was uninformative. In this case, the annotation of a subsequent hit from the same region was taken instead. Abbreviations are as follows: *Wolbachia* endosymbiont of *Culex quinquefasciatus, wCq*; *Wolbachia* endosymbiont of *Brugia malayi, wBm*; *Wolbachia* endosymbiont of *Drosophila simulans*, *wRi*; *Wolbachia* endosymbiont of *Drosophila melanogaster*, *wDm*; *Wolbachia* endosymbiont of *Onchocerca volvulus, wOv*; *Wolbachia* endosymbiont of *Dirofilaria immitis, wDi*. The average length of a sequence with homology to a *Wolbachia* gene was 158.9±82.6bp. The average percent identity of an *A. viteae* sequence to a *Wolbachia* gene was 78.0±6.0%, while the average percent identity to a nematode gene was 85.2±4.5%. The difference between the average size of a *Wolbachia* homolog and a nematode homolog was statistically significant according to Student’s t-test (p-value = 1.42 e-07).
