# Supplementary material for: Endosymbiont DNA in Endobacteria-Free Filarial Nematodes Indicates Ancient Horizontal Genetic Transfer
Source: PLoS One. 2010 Jun 9;5(6):e11029. doi: 10.1371/journal.pone.0011029 (PMC2882956; doi:10.1371/journal.pone.0011029)
Supplement: Table S2 — BLASTN annotation Onchocerca flexuosa genomic DNA fragments. BLASTN based annotation of all O. flexuosa contigs and read sequences that contain Wolbachia homologs with an e-value less than 1e-05. Annotation given is that of the top blast hit unless description of top hit was uninformative. In this case, the annotation of a subsequent hit from the same region was taken instead. Abbreviations are as follows: Wolbachia endosymbiont of Drosophila simulans, wRi; Wolbachia endosymbiont of Brugia malayi, wBm; Wolbachia endosymbiont of Onchocerca volvulus, wOv; Wolbachia endosymbiont of Culex quinquefasciatus, wCq; Wolbachia endosymbiont of Dirofilaria immitis, wDi; Wolbachia endosymbiont of Drosophila melanogaster, wDm. The average length of a sequence with homology to a Wolbachia gene was 173.6 plus or minus 191.8bp. The average percent identity of an O.flexuosa sequence to a Wolbachia gene was 80.6 plus or minus 6.0%, while the average percent identity to a nematode gene was 83.1 plus or minus 6.1%. This difference was statistically significant according to Student's t-test (p-value = .0014). (0.15 MB DOC) [file pone.0011029.s002.doc]

**Table S2.** BLASTN annotation *Onchocerca flexuosa* genomic DNA fragments.

| **Fragment #** | **Length (bp)** | **Locus Name** | **Best Annotation** | **Homolog Species** | **5' coord.** | **3' coord.** | **e-value** | **%ID** |
| --- | --- | --- | --- | --- | --- | --- | --- | --- |
| 001 | 1722 | wOf1a | [type IV secretion system protein VirB6, putative](http://www.ncbi.nlm.nih.gov/entrez/viewer.fcgi?val=225591853&db=Nucleotide&from=884124&to=887069&view=gbwithparts&RID=7EHRVUAY016) | *wRi* | 1 | 81 | 9.0E-11 | 81% |
|  |  | wOf1b | methionyl-tRNA synthetase, Na+/alanine symporter | *wBm* | 224 | 352 | 4.0E-21 | 79% |
|  |  | Of 1a | putative transcriptional activator ADA2, putative prohibitin | *Plasmodium falciparum* | 404 | 449 | 5.0E-08 | 93% |
|  |  | Of 1b.1 | hypothetical protein | *B. malayi* | 473 | 686 | 3.0E-68 | 87% |
|  |  | Of 1b.2 |  |  | 932 | 1200 | 3.0E-87 | 87% |
|  |  | Of 1b.3 |  |  | 1418 | 1525 | 1.0E-27 | 87% |
| 002 | 3502 | wOf2 | [ferredoxin, iron-sulfur cluster assembly system](http://www.ncbi.nlm.nih.gov/entrez/viewer.fcgi?val=225591853&db=Nucleotide&from=873712&to=874059&view=gbwithparts&RID=7EHRVUAY016) | *wRi* | 3388 | 3494 | 1.0E-21 | 80% |
| 003 | 2519 | Of3a | hypothetical protein partial mRNA | *B. malayi* | 171 | 263 | 5.0E-10 | 78% |
|  |  | Of3b | male EST sequence | *A. caninum* | 382 | 576 | 4.0E-36 | 80% |
|  |  | Of3b | adult female cDNA | *O. volvulus* | 782 | 1215 | 8.0E-70 | 74% |
|  |  | wOf3 | [lipoprotein releasing system transmembrane protein lolc](http://www.ncbi.nlm.nih.gov/entrez/viewer.fcgi?val=225591853&db=Nucleotide&from=1295163&to=1296392&view=gbwithparts&RID=B4ZUJ4F201N) | *wOv* | 1305 | 2519 | 0.0E+00 | 82% |
| 004 | 216 | wOf4 | [delta-aminolevulinic acid dehydratase](http://www.ncbi.nlm.nih.gov/entrez/viewer.fcgi?val=58418577&db=Nucleotide&from=476052&to=477047&view=gbwithparts&RID=7EHRVUAY016) | *wBm* | 1 | 184 | 1.0E-41 | 82% |
| 005 | 2453 | Of 5.1 | cDNA sequence BC017158 partial mRNA | *B. malayi* | 110 | 339 | 4.0E-91 | 86% |
|  |  | Of 5.2 |  |  | 899 | 1169 | 6.0E-64 | 80% |
|  |  | wOf5a | [4-Hydroxy-3-methylbut-2-enyl diphosphate reductase, IspH](http://www.ncbi.nlm.nih.gov/entrez/viewer.fcgi?val=58418577&db=Nucleotide&from=58809&to=59738&view=gbwithparts&RID=7EHRVUAY016) | *wBm* | 1893 | 2075 | 1.0E-21 | 75% |
|  |  | wOf5b | [acid phosphatase SurE](http://www.ncbi.nlm.nih.gov/entrez/viewer.fcgi?val=225591853&db=Nucleotide&from=1202041&to=1202793&view=gbwithparts&RID=9XUAZNE7014) | *wBm* | 2111 | 2413 | 7.0E-69 | 80% |
| 006 | 172 | wOf6 | [Na+/alanine symporter](http://www.ncbi.nlm.nih.gov/entrez/viewer.fcgi?val=225591853&db=Nucleotide&from=488612&to=489955&view=gbwithparts&RID=7EHRVUAY016) | *wRi* | 18 | 164 | 1.0E-34 | 83% |
| 007 | 3522 | Of7.1 | FERM domain partial mRNA | *B. malayi* | 6 | 910 | 0.0E+00 | 86% |
|  |  | Of7.2 |  |  | 1012 | 1089 | 6.0E-19 | 89% |
|  |  | Of7.3 |  |  | 1297 | 1464 | 1.0E-59 | 91% |
|  |  | Of7.4 |  |  | 2468 | 2572 | 8.0E-24 | 85% |
|  |  | Of7.5 |  |  | 2900 | 3113 | 6.0E-64 | 86% |
|  |  | wOf7 | [Na+/alanine symporter](http://www.ncbi.nlm.nih.gov/entrez/viewer.fcgi?val=225591853&db=Nucleotide&from=488612&to=489955&view=gbwithparts&RID=7EHRVUAY016) | *wRi* | 3356 | 3522 | 1.0E-53 | 89% |
| 008 | 2511 | wOf8 | [phosphoribosylglycinamide formyltransferase, putative](http://www.ncbi.nlm.nih.gov/entrez/viewer.fcgi?val=190356750&db=Nucleotide&from=541077&to=541643&view=gbwithparts&RID=7EHRVUAY016) | *wCq* | 3 | 105 | 4.0E-15 | 80% |
|  |  | Of8a.1 | hypothetical protein | *B. malayi* | 373 | 463 | 3.0E-17 | 84% |
|  |  | Of8a.2 |  |  | 761 | 879 | 4.0E-22 | 81% |
|  |  | Of8b | Ser/Thr protein phosphatase family protein | *B. malayi* | 2222 | 2354 | 1.0E-27 | 82% |
| 009 | 3504 | Of9a.1 | hypothetical protein | *B. malayi* | 930 | 1117 | 3.0E-36 | 79% |
|  |  | Of9a.2 |  |  | 1377 | 1540 | 3.0E-16 | 72% |
|  |  | Of9b | clone H29 microsatellite | *Ambystoma macrodactylum* | 1559 | 1606 | 5.0E-08 | 91% |
|  |  | Of9c | clone Pop1-2C5 | *Populus trichocarpa* | 3212 | 3293 | 6.0E-07 | 80% |
|  |  | wOf9 | [type IV secretion system protein VirD4](http://www.ncbi.nlm.nih.gov/entrez/viewer.fcgi?val=225591853&db=Nucleotide&from=8305&to=10323&view=gbwithparts&RID=7EHRVUAY016) | *wOv* | 3338 | 3429 | 4.0E-22 | 88% |
| 010 | 275 | wOf10 | [aspartyl-tRNA synthetase](http://www.ncbi.nlm.nih.gov/entrez/viewer.fcgi?val=190356750&db=Nucleotide&from=409770&to=411572&view=gbwithparts&RID=7EHRVUAY016) | *wCq* | 30 | 275 | 1.0E-41 | 74% |
| 011 | 251 | wOf11 | [guanylate kinase](http://www.ncbi.nlm.nih.gov/entrez/viewer.fcgi?val=58418577&db=Nucleotide&from=756340&to=756927&view=gbwithparts&RID=7ENFZ90B014) | *wBm* | 126 | 251 | 7.0E-21 | 79% |
| 013 | 4605 | wOf13a | [carbamoyl-phosphate synthase, large subunit](http://www.ncbi.nlm.nih.gov/entrez/viewer.fcgi?val=190356750&db=Nucleotide&from=980486&to=983704&view=gbwithparts&RID=7ENFZ90B014) | *wCq* | 1398 | 1579 | 2.0E-33 | 78% |
|  |  | wOf13b | [succinate dehydrogenase, cytochrome b556 subunit](http://www.ncbi.nlm.nih.gov/entrez/viewer.fcgi?val=190356750&db=Nucleotide&from=1137600&to=1137971&view=gbwithparts&RID=7ENFZ90B014) | *wCq* | 1733 | 1939 | 1.0E-35 | 77% |
|  |  | wOf13c.1 | [putative cell division protein FtsK](http://www.ncbi.nlm.nih.gov/entrez/viewer.fcgi?val=190356750&db=Nucleotide&from=206029&to=208140&view=gbwithparts&RID=7ENFZ90B014) | *wCq* | 1953 | 2032 | 3.0E-07 | 77% |
|  |  | wOf13c.2 |  |  | 2054 | 2270 | 1.0E-68 | 88% |
|  |  | wOf13d | [lipolytic enzyme, GDSL family](http://www.ncbi.nlm.nih.gov/entrez/viewer.fcgi?val=225591853&db=Nucleotide&from=1412860&to=1413840&view=gbwithparts&RID=98RVJ64J012) | *wRi* | 2303 | 2401 | 7.0E-10 | 76% |
|  |  | Of13.1 | helix-loop-helix DNA-binding domain containing protein | *B. malayi* | 4044 | 4326 | 6.0E-41 | 73% |
|  |  | Of13.2 |  |  | 4306 | 4473 | 2.0E-53 | 88% |
| 014 | 251 | wOf14 | [cytochrome b561](http://www.ncbi.nlm.nih.gov/entrez/viewer.fcgi?val=190356750&db=Nucleotide&from=690553&to=691074&view=gbwithparts&RID=7ENFZ90B014) | *wCq* | 67 | 145 | 5.0E-16 | 86% |
| 015 | 2973 | Of15.1 | myosin head containing protein partial mRNA | *B. malayi* | 706 | 861 | 6.0E-28 | 78% |
|  |  | Of15.2 |  |  | 1073 | 1208 | 4.0E-36 | 86% |
|  |  | Of15.3 |  |  | 1406 | 1549 | 7.0E-27 | 79% |
|  |  | wOf15 | [valyl-tRNA synthetase](http://www.ncbi.nlm.nih.gov/entrez/viewer.fcgi?val=190356750&db=Nucleotide&from=97610&to=100183&view=gbwithparts&RID=7ENFZ90B014) | *wCq* | 2771 | 2973 | 2.0E-52 | 83% |
| 016 | 7725 | Of16 | male EST sequence | *A. caninum* | 4138 | 4430 | 3.0E-17 | 69% |
|  |  | wOf16a | [putative oxidoreductase, 2-nitropropane dioxygenase family](http://www.ncbi.nlm.nih.gov/entrez/viewer.fcgi?val=225591853&db=Nucleotide&from=172110&to=173513&view=gbwithparts&RID=7ENFZ90B014) | *wRi* | 5984 | 6084 | 2.0E-15 | 80% |
|  |  | wOf16b | [arginyl-tRNA synthetase](http://www.ncbi.nlm.nih.gov/entrez/viewer.fcgi?val=225591853&db=Nucleotide&from=410332&to=412038&view=gbwithparts&RID=7ENFZ90B014) | *wRi* | 6088 | 6213 | 5.0E-16 | 76% |
|  |  | wOf16c | [DNA topoisomerase I](http://www.ncbi.nlm.nih.gov/entrez/viewer.fcgi?val=225591853&db=Nucleotide&from=1145928&to=1148378&view=gbwithparts&RID=7ENFZ90B014) | *wRi* | 6214 | 6338 | 2.0E-21 | 80% |
|  |  | wOf16d | [rod shape-determining protein RodA](http://www.ncbi.nlm.nih.gov/entrez/viewer.fcgi?val=225591853&db=Nucleotide&from=1370822&to=1371925&view=gbwithparts&RID=7ENFZ90B014) | *wRi* | 6348 | 6512 | 1.0E-43 | 84% |
|  |  | wOf16e | [midophosphoribosyltransferase](http://www.ncbi.nlm.nih.gov/entrez/viewer.fcgi?val=225591853&db=Nucleotide&from=1371951&to=1373336&view=gbwithparts&RID=7ENFZ90B014) | *wRi* | 6577 | 6638 | 8.0E-14 | 90% |
| 017 | 1124 | wOf17 | [parvulin-like peptidyl-prolyl isomerase, PPID](http://www.ncbi.nlm.nih.gov/entrez/viewer.fcgi?val=58418577&db=Nucleotide&from=688142&to=689950&view=gbwithparts&RID=7ENFZ90B014) | *wCq* | 1066 | 1119 | 2.0E-09 | 88% |
| 018 | 164 | wOf18 | [outer membrane protein/protective antigen OMA87](http://www.ncbi.nlm.nih.gov/entrez/viewer.fcgi?val=58418577&db=Nucleotide&from=73683&to=76019&view=gbwithparts&RID=7ENFZ90B014) | *wBm* | 100 | 164 | 9.0E-13 | 87% |
| 019 | 3075 | wOf19.1 | [DNA mismatch repair protein MutL](http://www.ncbi.nlm.nih.gov/entrez/viewer.fcgi?val=225591853&db=Nucleotide&from=1419589&to=1421406&view=gbwithparts&RID=7ENFZ90B014) | *wRi* | 2855 | 2962 | 7.0E-21 | 82% |
|  |  | wOf19.2 |  |  | 2997 | 3072 | 3.0E-12 | 84% |
| 020 | 193 | wOf20 | [putaive outer membrane protein](http://www.ncbi.nlm.nih.gov/entrez/viewer.fcgi?val=190356750&db=Nucleotide&from=1446900&to=1448060&view=gbwithparts&RID=7ENFZ90B014) | *wCq* | 67 | 153 | 6.0E-15 | 84% |
| 021 | 256 | wOf21 | [triosephosphate isomerase](http://www.ncbi.nlm.nih.gov/entrez/viewer.fcgi?val=190356750&db=Nucleotide&from=572297&to=572998&view=gbwithparts&RID=7GKUVEHE01S) | *wDi* | 41 | 210 | 2.0E-28 | 77% |
| 022 | 1955 | Of22.1 | sm protein partial mRNA | *B. malayi* | 213 | 338 | 8.0E-20 | 78% |
|  |  | Of22.2 |  |  | 826 | 1053 | 3.0E-45 | 78% |
|  |  | wOf22 | virD4 gene for type IV secretion system | *wOv* | 1768 | 1953 | 1.0E-62 | 87% |
| 023 | 1299 | wOf23 | [Asp-tRNAAsn/Glu-tRNAGln amidotransferase A subunit](http://www.ncbi.nlm.nih.gov/entrez/viewer.fcgi?val=58418577&db=Nucleotide&from=1000701&to=1002137&view=gbwithparts&RID=7GKUVEHE01S) | *wOv* | 45 | 200 | 1.0E-30 | 81% |
| 024 | 257 | wOf24 | [heat shock sigma factor RpoH](http://www.ncbi.nlm.nih.gov/entrez/viewer.fcgi?val=225591853&db=Nucleotide&from=1190187&to=1190999&view=gbwithparts&RID=7GKUVEHE01S) | *wOv* | 129 | 246 | 4.0E-30 | 86% |
| 025 | 258 | wOf25 | [multisubunit Na+/H+ antiporter, MnhD subunit](http://www.ncbi.nlm.nih.gov/entrez/viewer.fcgi?val=58418577&db=Nucleotide&from=65039&to=66544&view=gbwithparts&RID=7GKUVEHE01S) | *wBm* | 141 | 198 | 1.0E-12 | 91% |
| 026 | 274 | wOf26.1 | [rRNA-23S ribosomal RNA](http://www.ncbi.nlm.nih.gov/entrez/viewer.fcgi?val=225591853&db=Nucleotide&from=188532&to=191277&view=gbwithparts&RID=7GKUVEHE01S) | *wOv* | 20 | 179 | 1.0E-50 | 88% |
|  |  | wOf26.2 |  |  | 207 | 274 | 1.0E-12 | 86% |
| 027 | 233 | wOf27 | [heme exporter protein CcmA](http://www.ncbi.nlm.nih.gov/entrez/viewer.fcgi?val=225591853&db=Nucleotide&from=360017&to=360640&view=gbwithparts&RID=7GKUVEHE01S) | *wBm* | 170 | 229 | 5.0E-10 | 86% |
| 028 | 2859 | wOf28a | [membrane protein CvpA, putative](http://www.ncbi.nlm.nih.gov/entrez/viewer.fcgi?val=42410857&db=Nucleotide&from=117503&to=118093&view=gbwithparts&RID=7GKUVEHE01S) | *wDm* | 88 | 229 | 6.0E-41 | 87% |
|  |  | wOf28b | Onchocerca *Wolbachia* Sequence Fragment OW4 | *wOv* | 233 | 717 | 6.0E-123 | 79% |
|  |  | wOf28c | [alanyl-tRNA synthetase](http://www.ncbi.nlm.nih.gov/entrez/viewer.fcgi?val=225591853&db=Nucleotide&from=897437&to=900079&view=gbwithparts&RID=7GKUVEHE01S) | *wRi* | 793 | 910 | 6.0E-15 | 75% |
|  |  | Of28.1 | histidine acid phosphatase family protein partial | *B. malayi* | 1292 | 1406 | 6.0E-22 | 81% |
|  |  | Of28.2 |  |  | 1678 | 1818 | 2.0E-33 | 83% |
| 029 | 196 | wOf29 | Onchocerca *Wolbachia* Sequence Fragment OW2 | *wOv* | 44 | 180 | 2.0E-33 | 84% |
| 030 | 1923 | wOf30 | [ABC transporter, ATP-binding protein](http://www.ncbi.nlm.nih.gov/entrez/viewer.fcgi?val=225591853&db=Nucleotide&from=100614&to=101387&view=gbwithparts&RID=7GKUVEHE01S) | *wRi* | 1 | 46 | 7.0E-08 | 91% |
|  |  | Of30 | [hypothetical uncharacterized protein MG423](http://www.ncbi.nlm.nih.gov/entrez/viewer.fcgi?val=239984707&db=Nucleotide&from=240476&to=242158&view=gbwithparts&RID=7GKUVEHE01S) | *Mycoplasma conjunctivae* | 402 | 517 | 7.0E-08 | 72% |
| 031 | 123 | wOf31 | DNA polymerase III, beta subunit | *wCq* | 52 | 123 | 5.0E-18 | 90% |
| 032 | 3316 | wOf32 | [DNA polymerase III alpha subunit](http://www.ncbi.nlm.nih.gov/entrez/viewer.fcgi?val=58418577&db=Nucleotide&from=649784&to=653122&view=gbwithparts&RID=7GP6ADB7012) | *wBm* | 57 | 175 | 2.0E-30 | 86% |
|  |  | Of32a | zf-1 gene | *O. volvulus* | 612 | 837 | 2.0E-17 | 70% |
|  |  | Of32b | zinc finger 2 protein | *O. volvulus* | 1502 | 1644 | 4.0E-13 | 73% |
|  |  | Of32c | AmaD203 microsatellite sequence | *Ambystoma maculatum* | 2671 | 2739 | 3.0E-08 | 81% |
| 033 | 2269 | Of33 | AmaD315 microsatellite sequence | *Ambystoma maculatum* | 1468 | 1529 | 1.0E-07 | 82% |
|  |  | wOf33 | [translation elongation factor G](http://www.ncbi.nlm.nih.gov/entrez/viewer.fcgi?val=190356750&db=Nucleotide&from=607151&to=609226&view=gbwithparts&RID=7GP6ADB7012) | *wCq* | 2196 | 2268 | 4.0E-13 | 84% |
| 034 | 1772 | wOf34 | [phosphopantetheine adenylyltransferase](http://www.ncbi.nlm.nih.gov/entrez/viewer.fcgi?val=42410857&db=Nucleotide&from=513887&to=514393&view=gbwithparts&RID=7GP6ADB7012) | *wDm* | 100 | 223 | 7.0E-29 | 84% |
|  |  | Of34a | BAC clone CH251-433B9 | *Pan troglodytes* | 982 | 1169 | 1.0E-13 | 70% |
|  |  | Of34b | SETMAR gene | *Symphalangus syndactylus* | 1226 | 1496 | 9.0E-34 | 72% |
| 035 | 5594 | Of35a.1 | vacuolar H ATPase protein 16 | *B. malayi* | 849 | 1008 | 1.0E-56 | 91% |
|  |  | Of35a.2 |  |  | 1167 | 1316 | 1.0E-45 | 88% |
|  |  | Of35a.3 |  |  | 1379 | 1492 | 4.0E-26 | 84% |
|  |  | Of35a.4 |  |  | 1730 | 1889 | 1.0E-57 | 91% |
|  |  | Of35a.5 |  |  | 2189 | 2366 | 3.0E-59 | 89% |
|  |  | Of35a.6 |  |  | 2474 | 2574 | 1.0E-32 | 91% |
|  |  | Of35a.7 |  |  | 2665 | 2744 | 1.0E-18 | 87% |
|  |  | Of35a.8 |  |  | 3099 | 3223 | 1.0E-37 | 88% |
|  |  | wOf35 | [peptidase S66 family protein](http://www.ncbi.nlm.nih.gov/entrez/viewer.fcgi?val=190356750&db=Nucleotide&from=714947&to=715906&view=gbwithparts&RID=7GPUBS54012) | *wCq* | 3930 | 4108 | 2.0E-23 | 76% |
|  |  | Of35b | protein kinase domain containing protein | *B. malayi* | 5054 | 5253 | 7.0E-54 | 84% |
| 036 | 3218 | wOf36a.1 | [tRNA modification GTPase TrmE](http://www.ncbi.nlm.nih.gov/entrez/viewer.fcgi?val=42410857&db=Nucleotide&from=940143&to=941669&view=gbwithparts&RID=98X9MZEK01N) | *wDm* | 37 | 95 | 2.0E-09 | 88% |
|  |  | wOf36a.2 |  |  | 115 | 179 | 6.0E-10 | 86% |
|  |  | Of36a.1 | hypothetical protein | *B. malayi* | 233 | 330 | 1.0E-13 | 78% |
|  |  | wOf36b | [ABC-type Fe3+ transport system, permease component](http://www.ncbi.nlm.nih.gov/entrez/viewer.fcgi?val=58418577&db=Nucleotide&from=50473&to=52071&view=gbwithparts&RID=98X9MZEK01N) | *wBm* | 532 | 672 | 5.0E-05 | 68% |
|  |  | Of36a.2 | hypothetical protein | *B. malayi* | 1516 | 1626 | 3.0E-20 | 81% |
|  |  | Of36a.3 |  |  | 2773 | 2979 | 2.0E-28 | 74% |
| 037 | 3335 | Of37a.1 | deoxyribose-phosphate aldolase | *B. malayi* | 597 | 704 | 1.0E-25 | 85% |
|  |  | Of37a.2 |  |  | 1056 | 1232 | 1.0E-38 | 80% |
|  |  | Of37b.1 | pyridine nucleotide-disulphide oxidoreductase family protein | *B. malayi* | 2210 | 2353 | 2.0E-42 | 87% |
|  |  | Of37b.2 |  |  | 2577 | 2703 | 7.0E-35 | 87% |
|  |  | Of37b.3 |  |  | 2921 | 2997 | 1.0E-12 | 83% |
|  |  | Of37b.4 |  |  | 3084 | 3209 | 2.0E-35 | 87% |
|  |  | wOf37 | [ribosomal large subunit pseudouridine synthase C, putative](http://www.ncbi.nlm.nih.gov/entrez/viewer.fcgi?val=225591853&db=Nucleotide&from=355329&to=356252&view=gbwithparts&RID=7GPUBS54012) | *wRi* | 3257 | 3334 | 9.0E-15 | 84% |
| 038 | 2580 | wOf38 | [amidophosphoribosyltransferase](http://www.ncbi.nlm.nih.gov/entrez/viewer.fcgi?val=225591853&db=Nucleotide&from=1371951&to=1373336&view=gbwithparts&RID=7GPUBS54012) | *wOv* | 61 | 262 | 2.0E-60 | 86% |
|  |  | Of38.1 | DAN domain containing protein | *B. malayi* | 310 | 451 | 1.0E-38 | 85% |
|  |  | Of38.2 |  |  | 714 | 853 | 4.0E-38 | 85% |
|  |  | Of38.3 |  |  | 1044 | 1168 | 3.0E-33 | 86% |
| 039 | 1172 | wOf39 | [cell division septal protein](http://www.ncbi.nlm.nih.gov/entrez/viewer.fcgi?val=58418577&db=Nucleotide&from=749408&to=750166&view=gbwithparts&RID=80KG5AYH013) | *wBm* | 1117 | 1170 | 2.0E-09 | 89% |
| 040 | 190 | wOf40 | [ribosomal protein S13](http://www.ncbi.nlm.nih.gov/entrez/viewer.fcgi?val=58418577&db=Nucleotide&from=419487&to=419855&view=gbwithparts&RID=7GVYZUNP01S) | *wBm* | 42 | 190 | 1.0E-35 | 83% |
| 041 | 84 | wOf41 | [ribosomal protein L27](http://www.ncbi.nlm.nih.gov/entrez/viewer.fcgi?val=42410857&db=Nucleotide&from=124476&to=124745&view=gbwithparts&RID=7GZX5CHC014) | *wDm* | 4 | 50 | 5.0E-09 | 91% |
| 042 | 201 | wOf42a | [short-chain alcohol dehydrogenase family enzyme](http://www.ncbi.nlm.nih.gov/entrez/viewer.fcgi?val=58418577&db=Nucleotide&from=622220&to=622993&view=gbwithparts&RID=7H1GTHYZ012) | *wBm* | 1 | 112 | 1.0E-35 | 91% |
|  |  | wOf42b | [type IV secretory pathway, VirB6 components](http://www.ncbi.nlm.nih.gov/entrez/viewer.fcgi?val=58418577&db=Nucleotide&from=1058336&to=1060738&view=gbwithparts&RID=7H1GTHYZ012) | *wBm* | 118 | 196 | 8.0E-06 | 77% |
| 043 | 1889 | wOf43 | [hydrolase, alpha/beta fold family](http://www.ncbi.nlm.nih.gov/entrez/viewer.fcgi?val=225591853&db=Nucleotide&from=833785&to=834546&view=gbwithparts&RID=7H1GTHYZ012) | *wRi* | 33 | 187 | 1.0E-40 | 85% |
|  |  | Of43.1 | EF hand family protein | *B. malayi* | 1212 | 1401 | 6.0E-39 | 80% |
|  |  | Of43.2 |  |  | 1546 | 1619 | 2.0E-07 | 78% |
| 044 | 2550 | wOf44 | [1-deoxy-D-xylulose 5-phosphate reductoisomerase](http://www.ncbi.nlm.nih.gov/entrez/viewer.fcgi?val=225591853&db=Nucleotide&from=1012091&to=1013254&view=gbwithparts&RID=7H2FW64S014) | *wRi* | 53 | 83 | 2.0E-05 | 100% |
|  |  | Of44a.1 | ma315 microsatellite sequence | *Ambystoma maculatum* | 822 | 891 | 1.0E-16 | 90% |
|  |  | Of44a.2 |  |  | 1845 | 1903 | 1.0E-09 | 86% |
|  |  | Of44b | clone DKEY-191E17 | *Danio rerio* | 2480 | 2549 | 5.0E-08 | 82% |
| 045 | 55 | wOf45 | [dihydrodipicolinate reductase](http://www.ncbi.nlm.nih.gov/entrez/viewer.fcgi?val=58418577&db=Nucleotide&from=226355&to=227149&view=gbwithparts&RID=7H2WBTPS014) | *wBm* | 1 | 54 | 5.0E-05 | 81% |
| 046 | 249 | wOf46 | [dioxygenase related to 2-nitropropane dioxygenase](http://www.ncbi.nlm.nih.gov/entrez/viewer.fcgi?val=58418577&db=Nucleotide&from=330367&to=331749&view=gbwithparts&RID=7H1GTHYZ012) | *wBm* | 49 | 177 | 4.0E-35 | 86% |
| 047 | 154 | wOf47 | [molecular chaperone, HSP90 family, HtpG](http://www.ncbi.nlm.nih.gov/entrez/viewer.fcgi?val=58418577&db=Nucleotide&from=179189&to=181102&view=gbwithparts&RID=7H1GTHYZ012) | *wBm* | 11 | 148 | 4.0E-28 | 79% |
| 048 | 234 | wOf48 | [NAD-specific glutamate dehydrogenase](http://www.ncbi.nlm.nih.gov/entrez/viewer.fcgi?val=58418577&db=Nucleotide&from=233194&to=237927&view=gbwithparts&RID=7H1GTHYZ012) | *wRi* | 38 | 228 | 2.0E-44 | 81% |
| 049 | 3256 | Of49.1 | hypothetical protein | *B. malayi* | 1 | 151 | 5.0E-08 | 70% |
|  |  | Of49.2 |  |  | 372 | 429 | 2.0E-07 | 84% |
|  |  | Of49.3 |  |  | 1596 | 1719 | 1.0E-22 | 80% |
|  |  | wOf49a | [multisubunit Na+/H+ antiporter, MnhD subunit](http://www.ncbi.nlm.nih.gov/entrez/viewer.fcgi?val=58418577&db=Nucleotide&from=22569&to=24032&view=gbwithparts&RID=7H1GTHYZ012) | *wCq* | 2712 | 2876 | 3.0E-23 | 75% |
|  |  | wOf49b | [membrane-bound protoheme IX biogenesis protein, HemY](http://www.ncbi.nlm.nih.gov/entrez/viewer.fcgi?val=58418577&db=Nucleotide&from=627663&to=628826&view=gbwithparts&RID=7H1GTHYZ012) | *wCq* | 2928 | 3029 | 2.0E-19 | 82% |
|  |  | wOf49c | [FKBP-type peptidyl-prolyl cis-trans isomerase, tig](http://www.ncbi.nlm.nih.gov/entrez/viewer.fcgi?val=58418577&db=Nucleotide&from=729193&to=730536&view=gbwithparts&RID=7H1GTHYZ012) | *wCq* | 3089 | 3252 | 2.0E-39 | 82% |
| 050 | 258 | wOf50 | [type IV secretion system protein VirB4, putative](http://www.ncbi.nlm.nih.gov/entrez/viewer.fcgi?val=42410857&db=Nucleotide&from=1118862&to=1121228&view=gbwithparts&RID=7H1GTHYZ012) | *wDm* | 37 | 258 | 3.0E-48 | 79% |
| 051 | 3999 | Of51a | dehydrogenase, short chain protein 30 | *B. malayi* | 1 | 332 | 1.0E-106 | 86% |
|  |  | Of51b.1 | ShTK domain containing protein | *B. malayi* | 2218 | 2399 | 2.0E-28 | 77% |
|  |  | Of51b.2 |  |  | 2890 | 3022 | 7.0E-21 | 78% |
|  |  | wOf51 | [transcription elongation factor NusA](http://www.ncbi.nlm.nih.gov/entrez/viewer.fcgi?val=225591853&db=Nucleotide&from=1439317&to=1440879&view=gbwithparts&RID=7H4JBEH401S) | *wRi* | 3862 | 3994 | 3.0E-25 | 80% |
| 052 | 286 | wOf52 | [pyridine nucleotide-disulphide oxidoreductase family protein](http://www.ncbi.nlm.nih.gov/entrez/viewer.fcgi?val=225591853&db=Nucleotide&from=1003061&to=1004077&view=gbwithparts&RID=7H4JBEH401S) | *wRi* | 1 | 189 | 3.0E-19 | 72% |
| 053 | 2411 | wOf53a | [dimethyladenosine transferase](http://www.ncbi.nlm.nih.gov/entrez/viewer.fcgi?val=58418577&db=Nucleotide&from=527841&to=528626&view=gbwithparts&RID=7H4JBEH401S) | *wBm* | 1 | 98 | 9.0E-07 | 76% |
|  |  | wOf53b | [2-methylthioadenine synthetase](http://www.ncbi.nlm.nih.gov/entrez/viewer.fcgi?val=58418577&db=Nucleotide&from=128423&to=129658&view=gbwithparts&RID=7H4JBEH401S) | *wBm* | 172 | 512 | 1.0E-99 | 84% |
|  |  | wOf53c | phosphoribosylformylglycinamidine (FGAM) synthase | *wBm* | 516 | 626 | 6.0E-22 | 82% |
|  |  | wOf53d | [signal recognition particle GTPase](http://www.ncbi.nlm.nih.gov/entrez/viewer.fcgi?val=58418577&db=Nucleotide&from=63711&to=65054&view=gbwithparts&RID=7H4JBEH401S) | *wBm* | 725 | 848 | 1.0E-12 | 76% |
|  |  | Of53.1 | signal peptide peptidase family protein | *B. malayi* | 1432 | 1620 | 2.0E-28 | 75% |
|  |  | Of53.1 |  |  | 1981 | 2128 | 5.0E-23 | 77% |
| 054 | 2749 | wOf54 | [type IV secretion system protein VirB4, putative](http://www.ncbi.nlm.nih.gov/entrez/viewer.fcgi?val=225591853&db=Nucleotide&from=1235454&to=1237808&view=gbwithparts&RID=7H4JBEH401S) | *wRi* | 1 | 132 | 4.0E-30 | 83% |
|  |  | Of54a | BAC clone CH276-509J10 | *Pongo abelii* | 167 | 281 | 6.0E-09 | 72% |
|  |  | Of54b | BAC clone CH250-369M2 | *Macaca mulatta* | 336 | 545 | 4.0E-11 | 69% |
|  |  | Of54c | BAC RP11-54A6 | *Homo sapiens* | 1073 | 1236 | 1.0E-12 | 72% |
|  |  | Of54d | SL1 TOPO v1 adult cDNA | *D. immitis* | 1599 | 1882 | 6.0E-85 | 85% |
| 055 | 472 | wOf55 | [heme exporter protein CcmC](http://www.ncbi.nlm.nih.gov/entrez/viewer.fcgi?val=42410857&db=Nucleotide&from=323343&to=324053&view=gbwithparts&RID=8NWB418501N) | *wDm* | 1 | 290 | 2.0E-32 | 69% |
| 056 | 478 | Of56 | male EST sequence | *A. caninum* | 1 | 127 | 8.0E-17 | 77% |
|  |  | wOf56 | [predicted nucleotide kinase, YdiA family](http://www.ncbi.nlm.nih.gov/entrez/viewer.fcgi?val=58418577&db=Nucleotide&from=825671&to=826489&view=gbwithparts&RID=7H4JBEH401S) | *wBm* | 154 | 291 | 1.0E-28 | 81% |
|  |  | Of56 | male EST sequence | *A. caninum* | 318 | 486 | 1.0E-14 | 74% |
| 058 | 278 | wOf58.1 | [N utilization substance protein A](http://www.ncbi.nlm.nih.gov/entrez/viewer.fcgi?val=190356750&db=Nucleotide&from=1476012&to=1477565&view=gbwithparts&RID=7H4JBEH401S) | *wCq* | 1 | 62 | 2.0E-08 | 83% |
|  |  | wOf58.1 | [N utilization substance protein A](http://www.ncbi.nlm.nih.gov/entrez/viewer.fcgi?val=190356750&db=Nucleotide&from=1476012&to=1477565&view=gbwithparts&RID=7H4JBEH401S) |  | 161 | 265 | 5.0E-16 | 80% |
| 059 | 275 | wOf59 | [amino acid permease family protein](http://www.ncbi.nlm.nih.gov/entrez/viewer.fcgi?val=190356750&db=Nucleotide&from=786924&to=788210&view=gbwithparts&RID=7H4JBEH401S) | *wCq* | 54 | 195 | 7.0E-27 | 80% |
| 061 | 266 | wOf61 | [glutamine phosphoribosylpyrophosphate amidotransferase](http://www.ncbi.nlm.nih.gov/entrez/viewer.fcgi?val=58418577&db=Nucleotide&from=321593&to=322978&view=gbwithparts&RID=7K64E66X01N) | *wOv* | 14 | 250 | 2.0E-41 | 76% |
| 062 | 232 | wOf62 | [protoheme IX farnesyltransferase](http://www.ncbi.nlm.nih.gov/entrez/viewer.fcgi?val=225591853&db=Nucleotide&from=525986&to=526873&view=gbwithparts&RID=7K64E66X01N) | *wRi* | 1 | 225 | 2.0E-65 | 85% |
| 063 | 254 | wOf63 | [response regulator/GGDEF domain protein](http://www.ncbi.nlm.nih.gov/entrez/viewer.fcgi?val=42410857&db=Nucleotide&from=200878&to=202260&view=gbwithparts&RID=7K64E66X01N) | *wDm* | 1 | 245 | 2.0E-78 | 87% |
| 064 | 259 | wOf64 | [tRNA nucleotidyltransferase/poly(A) polymerase](http://www.ncbi.nlm.nih.gov/entrez/viewer.fcgi?val=58418577&db=Nucleotide&from=205874&to=207073&view=gbwithparts&RID=7K64E66X01N) | *wBm* | 71 | 258 | 1.0E-29 | 76% |
| 065 | 301 | wOf65 | Onchocerca *Wolbachia* sequence fragment OW3 | *wOv* | 47 | 209 | 1.0E-29 | 79% |
| 066 | 257 | wOf66 | cell cycle protein (ftsZ) gene | *wOv* | 2 | 183 | 2.0E-40 | 80% |
| 067 | 253 | wOf67 | [phosphatidylserine decarboxylase](http://www.ncbi.nlm.nih.gov/entrez/viewer.fcgi?val=225591853&db=Nucleotide&from=1175365&to=1176057&view=gbwithparts&RID=7K64E66X01N) | *wRi* | 21 | 162 | 1.0E-31 | 83% |
| 068 | 4116 | Of68a | histone H3 | *B. malayi* | 224 | 700 | 1.0E-131 | 80% |
|  |  | Of68b | [mitf, isoform A, mitf, isoform B](http://www.ncbi.nlm.nih.gov/entrez/viewer.fcgi?val=112980799&db=Nucleotide&from=1219641&to=1226517&view=gbwithparts&RID=7K72A3SJ01S) | *D. melanogaster* | 924 | 1207 | 4.0E-19 | 75% |
|  |  | Of68c | linker histone H1 and H5 family protein | *B. malayi* | 1808 | 2386 | 1.0E-100 | 74% |
|  |  | Of68d | histone H2B 2 | *B. malayi* | 2408 | 2469 | 4.0E-06 | 82% |
|  |  | wOf68a | [hypothetical protein](http://www.ncbi.nlm.nih.gov/entrez/viewer.fcgi?val=225591853&db=Nucleotide&from=1214465&to=1214743&view=gbwithparts&RID=9B2FCVP2016) | *wRi* | 2686 | 2750 | 8.0E-05 | 80% |
|  |  | wOf68b | glutamyl-tRNA amidotransferase, ankyrin repeat protein | *wDm* | 3040 | 3432 | 4.0E-31 | 68% |
|  |  | wOf68c | [predicted membrane GTPase involved in stress response](http://www.ncbi.nlm.nih.gov/entrez/viewer.fcgi?val=58418577&db=Nucleotide&from=81876&to=83705&view=gbwithparts&RID=7K72A3SJ01S) | *wBm* | 3602 | 4115 | 2.0E-80 | 73% |
| 069 | 5266 | Of69.1 | egg laying defective protein 4, isoform c | *B. malayi* | 85 | 216 | 8.0E-52 | 95% |
|  |  | Of69.2 |  |  | 566 | 711 | 2.0E-52 | 92% |
|  |  | Of69.3 |  |  | 854 | 1089 | 6.0E-85 | 90% |
|  |  | Of69.4 |  |  | 1220 | 1397 | 6.0E-66 | 92% |
|  |  | Of69.5 |  |  | 1567 | 1748 | 4.0E-68 | 92% |
|  |  | Of69.6 |  |  | 1918 | 2157 | 4.0E-62 | 82% |
|  |  | wOf69 | TPR domain-containing protein gene | *wDi* | 5120 | 5265 | 1.0E-23 | 77% |
| 070 | 6909 | Of70.1 | molting L3 larval transcript | *O. volvulus* | 1767 | 2074 | 9.0E-49 | 75% |
|  |  | wOf70 | [outer membrane protein TolC, putative](http://www.ncbi.nlm.nih.gov/entrez/viewer.fcgi?val=225591853&db=Nucleotide&from=62867&to=64090&view=gbwithparts&RID=7K64E66X01N) | *wBm* | 2739 | 4411 | 2.0E-78 | 64% |
|  |  | Of70.2 | molting L3 larval transcript | *O. volvulus* | 5486 | 5627 | 5.0E-33 | 85% |
|  |  | Of70.3 |  |  | 5675 | 5904 | 2.0E-31 | 74% |
| 071 | 548 | wOf71.1 | [trypsin-like serine protease](http://www.ncbi.nlm.nih.gov/entrez/viewer.fcgi?val=58418577&db=Nucleotide&from=1011537&to=1013027&view=gbwithparts&RID=8Y14SC3F01S) | *wOv* | 47 | 164 | 2.0E-18 | 79% |
|  |  | wOf71.2 | [trypsin-like serine protease](http://www.ncbi.nlm.nih.gov/entrez/viewer.fcgi?val=58418577&db=Nucleotide&from=1011537&to=1013027&view=gbwithparts&RID=8Y14SC3F01S) |  | 198 | 547 | 8.0E-56 | 72% |
| 072 | 3684 | wOf72 | [ATP-dependent Zn protease, HflB](http://www.ncbi.nlm.nih.gov/entrez/viewer.fcgi?val=58418577&db=Nucleotide&from=189447&to=191276&view=gbwithparts&RID=7KCCDW9901S) | *wBm* | 88 | 590 | 6.0E-135 | 80% |
|  |  | Of72a | BAC clone RP43-10A14 | *Pan troglodytes* | 1195 | 1242 | 4.0E-10 | 93% |
|  |  | Of72b.1 | actin family protein | *B. malayi* | 1405 | 1543 | 4.0E-29 | 82% |
|  |  | Of72b.2 |  |  | 1701 | 1851 | 3.0E-44 | 87% |
|  |  | Of72b.3 |  |  | 2175 | 2397 | 7.0E-77 | 89% |
|  |  | Of72b.4 |  |  | 2632 | 2727 | 7.0E-20 | 84% |
|  |  | Of72b.5 |  |  | 3026 | 3176 | 4.0E-36 | 83% |
|  |  | Of72b.6 |  |  | 3379 | 3474 | 2.0E-28 | 90% |
|  |  | Of72b.7 |  |  | 3574 | 3630 | 9.0E-19 | 86% |
| 073 | 255 | wOf73 | [3-octaprenyl-4-hydroxybenzoate carboxy-lyase](http://www.ncbi.nlm.nih.gov/entrez/viewer.fcgi?val=42410857&db=Nucleotide&from=1255384&to=1256862&view=gbwithparts&RID=7KCCDW9901S) | *wDm* | 92 | 254 | 5.0E-09 | 69% |
| 074 | 184 | wOf74 | [putative outer membrane protein](http://www.ncbi.nlm.nih.gov/entrez/viewer.fcgi?val=225591853&db=Nucleotide&from=169632&to=170804&view=gbwithparts&RID=7KCCDW9901S) | *wRi* | 50 | 184 | 1.0E-22 | 79% |
| 075 | 203 | wOf75 | [ribosomal protein L6](http://www.ncbi.nlm.nih.gov/entrez/viewer.fcgi?val=225591853&db=Nucleotide&from=545483&to=546028&view=gbwithparts&RID=7KCCDW9901S) | *wRi* | 9 | 80 | 4.0E-11 | 84% |
|  |  | Of75 | hypothetical protein LOC100118561 | *Nasonia vitripennis* | 85 | 128 | 8.0E-07 | 90% |
| 076 | 2658 | wOf76a | [phosphate ABC transporter, ATP-binding protein](http://www.ncbi.nlm.nih.gov/entrez/viewer.fcgi?val=225591853&db=Nucleotide&from=451590&to=452348&view=gbwithparts&RID=7KCCDW9901S) | *wDi* | 33 | 262 | 5.0E-73 | 87% |
|  |  | Of76a | EST from tissues | *Petromyzon marinus* | 262 | 371 | 3.0E-18 | 76% |
|  |  | Of76b | cDNA clone CAAA2826 | *Petromyzon marinus* | 594 | 1287 | 0.0E+00 | 84% |
|  |  | Of76c.1 | OFAA-aaa59g11.g1 EST | *O. flexuosa* | 1284 | 1401 | 6.0E-15 | 79% |
|  |  | Of76c.2 |  |  | 1438 | 1575 | 1.0E-16 | 77% |
|  |  | wOf76b | [DNA processing chain A](http://www.ncbi.nlm.nih.gov/entrez/viewer.fcgi?val=225591853&db=Nucleotide&from=158965&to=160053&view=gbwithparts&RID=7KCCDW9901S) | *wRi* | 2429 | 2655 | 8.0E-13 | 69% |
| 077 | 278 | wOf77 | [4-diphosphocytidyl-2C-methyl-D-erythritol kinase](http://www.ncbi.nlm.nih.gov/entrez/viewer.fcgi?val=190356750&db=Nucleotide&from=181496&to=182362&view=gbwithparts&RID=7KCCDW9901S) | *wCq* | 76 | 242 | 8.0E-13 | 72% |
| 078 | 2506 | wOf78 | OrpB and HemE genes | *wAv* | 5 | 165 | 1.0E-23 | 76% |
|  |  | Of78a | AIAC-aaa96d06.g1 male EST | *A. caninum* | 168 | 373 | 7.0E-33 | 77% |
|  |  | Of78b | AIAE-aab30e03.b1 female EST | *A. caninum* | 407 | 701 | 2.0E-116 | 92% |
|  |  | Of78c.1 | AIAC-aaa33h07.g1 male EST | *A. caninum* | 721 | 847 | 2.0E-15 | 77% |
|  |  | Of78c.2 |  |  | 866 | 1360 | 6.0E-154 | 84% |
| 079 | 269 | wOf79 | [DNA-directed RNA polymerase, beta/beta' subunits](http://www.ncbi.nlm.nih.gov/entrez/viewer.fcgi?val=190356750&db=Nucleotide&from=594197&to=602716&view=gbwithparts&RID=7KCCDW9901S) | *wCq* | 65 | 234 | 4.0E-10 | 71% |
| 080 | 1884 | wOf80 | [cytochrome b561, putative](http://www.ncbi.nlm.nih.gov/entrez/viewer.fcgi?val=225591853&db=Nucleotide&from=1232044&to=1232565&view=gbwithparts&RID=7KCCDW9901S) | *wRi* | 42 | 187 | 4.0E-11 | 71% |
|  |  | Of80 | molting L3 larval cDNA | *O. volvulus* | 897 | 1111 | 3.0E-61 | 86% |
| 081 | 263 | wOf81 | [dihydroorotase](http://www.ncbi.nlm.nih.gov/entrez/viewer.fcgi?val=225591853&db=Nucleotide&from=223884&to=225209&view=gbwithparts&RID=7KMZHDBC01N) | *wRi* | 152 | 261 | 2.0E-14 | 78% |
| 082 | 271 | wOf82 | [ATP-binding subunit of Clp protease, DnaK/DnaJ chaperones](http://www.ncbi.nlm.nih.gov/entrez/viewer.fcgi?val=58418577&db=Nucleotide&from=230606&to=233167&view=gbwithparts&RID=7KMZHDBC01N) | *wBm* | 24 | 119 | 5.0E-16 | 81% |
| 083 | 224 | wOf83 | [DNA mismatch repair protein MutL](http://www.ncbi.nlm.nih.gov/entrez/viewer.fcgi?val=225591853&db=Nucleotide&from=1419589&to=1421406&view=gbwithparts&RID=7KMZHDBC01N) | *wRi* | 10 | 110 | 5.0E-10 | 76% |
| 084 | 158 | wOf84 | [preprotein translocase subunit YidC](http://www.ncbi.nlm.nih.gov/entrez/viewer.fcgi?val=58418577&db=Nucleotide&from=242694&to=244412&view=gbwithparts&RID=7KMZHDBC01N) | *wBm* | 25 | 145 | 1.0E-10 | 73% |
| 085 | 235 | wOf85 | [ribosomal protein L1](http://www.ncbi.nlm.nih.gov/entrez/viewer.fcgi?val=190356750&db=Nucleotide&from=603734&to=604387&view=gbwithparts&RID=7KMZHDBC01N) | *wCq* | 150 | 231 | 2.0E-14 | 85% |
| 086 | 2775 | wOf86 | [Amino acid transporter](http://www.ncbi.nlm.nih.gov/entrez/viewer.fcgi?val=58418577&db=Nucleotide&from=55843&to=57120&view=gbwithparts&RID=7KMZHDBC01N) | [*wBm*](http://www.ncbi.nlm.nih.gov/entrez/viewer.fcgi?val=58418577&db=Nucleotide&from=55843&to=57120&view=gbwithparts&RID=7KMZHDBC01N) | 4 | 99 | 8.0E-14 | 80% |
|  |  | Of86a.1 | zgc:92856 partial mRNA | *B. malayi* | 290 | 445 | 7.0E-53 | 90% |
|  |  | Of86a.2 |  |  | 683 | 802 | 7.0E-34 | 88% |
|  |  | Of86b | hypothetical protein | *B. malayi* | 2329 | 2539 | 5.0E-67 | 87% |
| 087 | 150 | wOf87 | secreted protein with pentapeptide repeats | *wBm* | 18 | 121 | 2.0E-21 | 83% |
| 088 | 2786 | wOf88 | [DNA polymerase I](http://www.ncbi.nlm.nih.gov/entrez/viewer.fcgi?val=190356750&db=Nucleotide&from=1175516&to=1178044&view=gbwithparts&RID=7KMZHDBC01N) | *wCq* | 4 | 488 | 1.0E-75 | 74% |
|  |  | Of88.1 | hypothetical protein | *B. malayi* | 576 | 684 | 2.0E-40 | 95% |
|  |  | Of88.2 |  |  | 802 | 987 | 6.0E-66 | 90% |
|  |  | Of88.3 |  |  | 1080 | 1181 | 2.0E-27 | 87% |
|  |  | Of88.4 |  |  | 1512 | 1732 | 2.0E-78 | 90% |
|  |  | Of88.5 |  |  | 1880 | 2053 | 5.0E-54 | 87% |
|  |  | Of88.6 |  |  | 2305 | 2549 | 1.0E-100 | 93% |
| 089 | 3175 | Of89a | AIAE-aaa05b11.b1 female EST | *A. caninum* | 859 | 1160 | 2.0E-92 | 83% |
|  |  | Of89b |  |  | 1225 | 1420 | 8.0E-33 | 76% |
|  |  | Of89c | OFAA-aaa63f08.b1 EST | *O. flexuosa* | 1526 | 2156 | 0.0E+00 | 94% |
|  |  | wOf89.1 | [F0F1-type ATP synthase, alpha subunit, delta subunit](http://www.ncbi.nlm.nih.gov/entrez/viewer.fcgi?val=58418577&db=Nucleotide&from=415063&to=416604&view=gbwithparts&RID=7KMZHDBC01N) | *wRi* | 2788 | 2973 | 1.0E-30 | 77% |
|  |  | wOf89.2 |  |  | 3035 | 3175 | 8.0E-14 | 75% |
| 090 | 193 | wOf90 | [Zn-dependent carboxypeptidase](http://www.ncbi.nlm.nih.gov/entrez/viewer.fcgi?val=225591853&db=Nucleotide&from=1322602&to=1324023&view=gbwithparts&RID=7KMZHDBC01N) | *wRi* | 46 | 128 | 3.0E-12 | 82% |
| 091 | 264 | wOf91 | [DNA processing chain A](http://www.ncbi.nlm.nih.gov/entrez/viewer.fcgi?val=190356750&db=Nucleotide&from=1457783&to=1458868&view=gbwithparts&RID=7KR62HHN016) | *wCq* | 3 | 120 | 1.0E-12 | 75% |
| 092 | 258 | wOf92 | [alanyl-tRNA synthetase](http://www.ncbi.nlm.nih.gov/entrez/viewer.fcgi?val=58418577&db=Nucleotide&from=453110&to=455737&view=gbwithparts&RID=7KR62HHN016) | *wBm* | 112 | 220 | 2.0E-16 | 80% |
| 093 | 264 | wOf93 | CDP-diacylglycerol-serine O-phosphatidyltransferase, putative | *wRi* | 144 | 218 | 8.0E-08 | 80% |
| 094 | 2662 | wOf94 | [putative Band 7 family membrane protein](http://www.ncbi.nlm.nih.gov/entrez/viewer.fcgi?val=190356750&db=Nucleotide&from=1289584&to=1290453&view=gbwithparts&RID=7KR62HHN016) | *wCq* | 51 | 294 | 4.0E-30 | 74% |
|  |  | Of94 | thiol-specific antioxidant (TSA) | *O. volvulus* | 301 | 472 | 1.0E-42 | 79% |
| 095 | 1385 | wOf95a | [ribonuclease H](http://www.ncbi.nlm.nih.gov/entrez/viewer.fcgi?val=190356750&db=Nucleotide&from=76670&to=77107&view=gbwithparts&RID=7KRPMYWE01S) | *wCq* | 47 | 370 | 2.0E-62 | 76% |
|  |  | wOf95b | [putative oxidoreductase](http://www.ncbi.nlm.nih.gov/entrez/viewer.fcgi?val=190356750&db=Nucleotide&from=1097731&to=1099290&view=gbwithparts&RID=7KRPMYWE01S) | *wCq* | 376 | 614 | 2.0E-24 | 72% |

BLASTN based annotation of all *O. flexuosa* contigs and read sequences that contain *Wolbachia* homologs with an e-value less than 1e-05. Annotation given is that of the top blast hit unless description of top hit was uninformative. In this case, the annotation of a subsequent hit from the same region was taken instead. Abbreviations are as follows: *Wolbachia* endosymbiont of *Drosophila simulans*, *wRi*; *Wolbachia* endosymbiont of *Brugia malayi, wBm*; *Wolbachia* endosymbiont of *Onchocerca volvulus, wOv*; *Wolbachia* endosymbiont of *Culex quinquefasciatus, wCq*; *Wolbachia* endosymbiont of *Dirofilaria immitis, wDi; Wolbachia* endosymbiont of *Drosophila melanogaster*, *wDm*. The average length of a sequence with homology to a *Wolbachia* gene was 173.6±191.8bp. The average percent identity of an *O.flexuosa* sequence to a *Wolbachia* gene was 80.6±6.0%, while the average percent identity to a nematode gene was 83.1±6.1%. This difference was statistically significant according to Student’s t-test (p-value = .0014).
