# Supplementary material for: Endosymbiont DNA in Endobacteria-Free Filarial Nematodes Indicates Ancient Horizontal Genetic Transfer
Source: PLoS One. 2010 Jun 9;5(6):e11029. doi: 10.1371/journal.pone.0011029 (PMC2882956; doi:10.1371/journal.pone.0011029)
Supplement: Table S3 — BLASTX annotation of Acanthocheilonema viteae genomic DNA fragments. BLASTX based annotation of all A. viteae contigs containing Wolbachia homologs with a BLASTN e-value less than 1e-05. All hits to Wolbachia genes by BLASTX were recorded, regardless of e-value. Abbreviations are as follows: Wolbachia endosymbiont of Culex quinquefasciatus, wCq; Wolbachia endosymbiont of Drosophila simulans, wRi; Wolbachia endosymbiont of Brugia malayi, wBm; Wolbachia endosymbiont of Drosophila willistoni, wDw; Wolbachia endosymbiont of Muscidifurax uniraptor, wMu; Wolbachia endosymbiont of Onchocerca volvulus, wOv; Wolbachia endosymbiont of Armadillium vulgare, wAv. The average length of a sequence with homology to a Wolbachia protein was 123.8 plus or minus 73.5bp. The average percent ID to a Wolbachia protein was 62.3 plus or minus 13.8%. According to Student's t-test, this is significantly lower than the average percent identity to a nematode protein, 79.1 plus or minus 15.6% (p-value = .0001). The Student's t-test indicates that the average percent identity to a Wolbachia protein is also significantly lower than the percent identity of a sequence to a Wolbachia gene on the nucleotide level (p-value = 8e-10). (0.13 MB DOC) [file pone.0011029.s003.doc]

**Table S3.** BLASTX annotation of *Acanthocheilonema viteae* genomic DNA fragments

| **Contig #** | **Length (bp)** | **Locus Name** | **Best Annotation** | **Homologs Species** | **5' coord** | **3' coord** | **e-value** | **%ID** | **Reading Frame** | **# stop codons** |
| --- | --- | --- | --- | --- | --- | --- | --- | --- | --- | --- |
| 187 | 735 | wAv187 | disulfide bond formation protein, DsbB family | *wCq* | 79 | 216 | 2.00E-05 | 51% | 1 | 0 |
|  |  | Av187.1 | pao retrotransposon peptidase family protein | *Brugia malayi* | 353 | 481 | 7.00E-24 | 65% | -3 | 0 |
|  |  | Av187.2 |  |  | 478 | 708 | 7.00E-24 | 44% | -1 | 0 |
| 2452 | 1054 |  | no hit |  |  |  |  |  |  |  |
| 3046 | 978 | wAv3046.1 | isoleucyl-tRNA synthetase | *wCq* | 413 | 499 | 1.00E-06 | 75% | 2 | 0 |
|  |  | wAv3046.2 |  |  | 502 | 540 | 1.00E-06 | 76% | 1 | 0 |
| 4552 | 455 | wAv4552.1 | TldD protein | *wCq* | 181 | 300 | 1.5 | 50% | 1 | 0 |
|  |  | wAv4552.2 | TldD protein | *wRi* | 302 | 451 | 8.00E-10 | 66% | -2 | 1 |
| 4766 | 903 | Av4766 | hypothetical protein Bm1_04445 | *Brugia malayi* | 269 | 319 | 1.00E-01 | 82% | 2 | 0 |
|  |  | wAv4776 | hypothetical protein WPa_0771 | *wCq* | 534 | 626 | 3.00E-04 | 67% | 3 | 0 |
|  |  | Av4766 | hypothetical protein Bm1_04445 | *Brugia malayi* | 712 | 903 | 4.00E-24 | 82% | 1 | 1 |
| 5106 | 2297 | Av5106 | hypothetical protein Bm1_03055 | *Brugia malayi* | 771 | 998 | 0.001 | 38% | 3 | 0 |
| 9153 | 1261 | Av9153.1 | hypothetical protein | *Brugia malayi* | 1 | 171 | 3.00E-14 | 72% | -2 | 0 |
|  |  | Av9153.2 |  |  | 336 | 419 | 0.004 | 78% | -3 | 1 |
|  |  | Av9153.3 |  |  | 703 | 939 | 8.00E-29 | 94% | -2 | 0 |
|  |  | wAv9135 | DNA polymerase III, gamma/tau subunit | *wBm* | 1012 | 1260 | 9.00E-20 | 63% | -2 | 2 |
| 9497 | 785 | wAv9497 | penicillin-binding protein | *wDw* | 3 | 197 | 1.00E-11 | 59% | 3 | 2 |
|  |  | Av9497.1 | Protein kinase domain containing protein | *Brugia malayi* | 268 | 462 | 2.00E-23 | 84% | -3 | 0 |
|  |  | Av9497.2 |  |  | 542 | 673 | 5.00E-06 | 95% | -2 | 0 |
| 9524 | 933 | wAv9524a.1 | methionyl-tRNA synthetase | *wCq* | 64 | 129 | 4.00E-04 | 63% | -1 | 0 |
|  |  | wAv9524a.2 |  |  | 209 | 448 | 4.00E-04 | 47% | -3 | 3 |
|  |  | wAv9524b.1 | phenylalanyl-tRNA synthetase, beta subunit | *wCq* | 618 | 758 | 1.00E-05 | 49% | -2 | 1 |
|  |  | wAv9524b.2 |  |  | 764 | 820 | 1.00E-05 | 52% | -3 | 0 |
|  |  | wAv9524b.3 |  |  | 820 | 897 | 1.00E-05 | 57% | -1 | 0 |
| 10096 | 1382 | wAv10096.1 | ATP-dependent exoDNAse, RecB | *wDw* | 592 | 699 | 8.00E-15 | 75% | 1 | 0 |
|  |  | wAv10096.2 |  |  | 711 | 884 | 8.00E-15 | 50% | 3 | 1 |
|  |  | wAv10096.3 |  |  | 983 | 1090 | 6.9 | 54% | 2 | 2 |
|  |  | wAv10096.4 | ATP-dependent exoDNAse, RecB | *wBm* | 1090 | 1224 | 8.00E-04 | 33% | 1 | 1 |
| 10682 | 241 | wAv10682 | 4-hydroxy-3-methylbut-2-enyl diphosphate reductase | *wRi* | 1 | 177 | 2.00E-05 | 54% | -2 | 1 |
| 11045 | 1235 | Av11045.1 | MGC80088 protein | *Brugia malayi* | 427 | 648 | 3.00E-46 | 82% | -3 | 0 |
|  |  | Av11045.2 |  |  | 746 | 868 | 3.00E-46 | 97% | -2 | 0 |
|  |  |  |  |  | 1075 | 1233 | 2.00E-20 | 96% | -3 | 0 |
| 11910 | 483 | wAv11910.1 | actin-like ATPase involved in cell morphogenesis, MreB | *wBm* | 42 | 311 | 3.00E-19 | 50% | 3 | 5 |
|  |  | wAv11910.2 |  |  | 350 | 466 | 3.00E-19 | 56% | 2 | 1 |
| 13336 | 1185 | Av13336.1 | DOMON domain containing protein | *Brugia malayi* | 2 | 352 | 2.00E-39 | 70% | 2 | 2 |
|  |  | Av13336.2 |  |  | 933 | 1100 | 7.00E-19 | 78% | 3 | 0 |
| 16332 | 607 | wAv16332.1 | phage uncharacterized protein | *wMu* | 183 | 236 | 8.00E-06 | 72% | -3 | 0 |
|  |  | wAv16332.2 |  |  | 256 | 492 | 8.00E-06 | 56% | -2 | 2 |
| 16679 | 1339 |  | no hit |  |  |  |  |  |  |  |
| 16952 | 890 | wAv16952 | DNA-directed RNA polymerase | *wBm* | 8 | 169 | 9.00E-13 | 68% | 2 | 1 |
| 19080 | 1434 | Av19080a.1 | SH2 domain containing protein | *Brugia malayi* | 130 | 291 | 8.00E-14 | 70% | 1 | 0 |
|  |  | Av19080a.2 |  |  | 510 | 641 | 4.00E-13 | 86% | 3 | 0 |
|  |  | Av19080b | signal transduction protein lnk-realted, putative | *Ixodes scapularis* | 1161 | 1277 | 8.00E-06 | 62% | 3 | 0 |
|  |  | wAv19080 | 2-oxoglutarate dehydrogenase E1 component | *wRi* | 1289 | 1426 | 4.00E-06 | 63% | 2 | 0 |
| 20032 | 669 |  | no hit |  |  |  |  |  |  |  |
| 21852 | 241 | wAv21852 | transketolase | *wBm* | 112 | 225 | 2.00E-06 | 72% | -2 | 0 |
| 22549 | 242 |  | no hit |  |  |  |  |  |  |  |
| 22942 | 608 |  | no hit |  |  |  |  |  |  |  |
| 24238 | 691 | wAv24238.1 | NADH dehydrogenase I, subunit G | *wRi* | 103 | 219 | 6.00E-10 | 64% | -2 | 0 |
|  |  | wAv24238.2 |  |  | 251 | 283 | 6.00E-10 | 81% | -1 | 0 |
|  |  | Av24238 | RNA polymerase II | *Brugia malayi* | 300 | 494 | 2.00E-24 | 80% | 3 | 0 |
| 24716 | 1676 | Av24716.1 | transcription factor Ash2 | *Brugia malayi* | 1238 | 1327 | 1.00E-24 | 86% | 2 | 0 |
|  |  | Av24716.2 |  |  | 1440 | 1547 | 1.00E-24 | 97% | 3 | 0 |
| 25554 | 240 |  | no hit |  |  |  |  |  |  |  |
| 28511 | 796 |  | no hit |  |  |  |  |  |  |  |
| 29055 | 465 | wAv29055.1 | undecaprenyl pyrophosphate synthase | *wBm* | 8 | 73 | 3.00E-14 | 59% | -3 | 0 |
|  |  | wAv29055.2 |  |  | 93 | 245 | 3.00E-14 | 58% | -2 | 0 |
|  |  | Av29055 | electron transfer flavoprotein-ubiquinone oxidoreductase | *Brugia malayi* | 351 | 464 | 8.00E-14 | 94% | -2 | 0 |
| 30005 | 282 | wAv30005.1 | excinuclease ABC subunit | *wBm* | 190 | 237 | 1.3 | 68% | 1 | 0 |
|  |  | wAv30005.2 |  |  | 227 | 280 | 1.3 | 70% | 2 | 0 |
| 30574 | 241 |  | no hit |  |  |  |  |  |  |  |
| 31107 | 585 | Av31107 | FRG1 protein homolog | *Brugia malayi* | 417 | 533 | 3.00E-08 | 71% | 3 | 0 |
| 31515 | 225 | wAv31515.1 | pseudouridylate synthase, 23S RNA-specific | *wBm* | 12 | 98 | 0.001 | 76% | 3 | 1 |
|  |  | wAv31515.2 |  |  | 98 | 142 | 0.001 | 73% | 2 | 1 |
| 31988 | 174 |  | no hit |  |  |  |  |  |  |  |
| 35539 | 223 |  | no hit |  |  |  |  |  |  |  |
| 36441 | 380 |  | no hit |  |  |  |  |  |  |  |
| 38375 | 1406 |  | no hit |  |  |  |  |  |  |  |
| 38543 | 467 | wAv38543 | methylase of polypeptide chain release factor | *wBm* | 44 | 256 | 1.00E-06 | 46% | 2 | 2 |
| 41791 | 217 | wAv41791.1 | hypothetical protein WUni_005190 | *wMu* | 82 | 135 | 0.074 | 83% | -2 | 1 |
|  |  | wAv41791.1 |  |  | 173 | 211 | 0.074 | 84% | -1 | 0 |
| 42190 | 366 | wAv41290 | IMP dehydrogenase, GuaB | *wBm* | 128 | 211 | 0.7 | 57% | -3 | 0 |
| 46345 | 259 | wAv46345 | cytochrome b subunit of the bc complex | *wBm* | 2 | 61 | 7.00E-03 | 80% | -1 | 0 |
| 48068 | 229 |  | no hit |  |  |  |  |  |  |  |
| 52396 | 242 |  | no hit |  |  |  |  |  |  |  |
| 55693 | 437 | wAv55693.1 | ATP-dependent Clp protease, ATP-binding subunit ClpB | *wBm* | 170 | 289 | 6.00E-07 | 67% | -2 | 0 |
|  |  | wAv55693.2 | ATP-dependent Clp protease, ATP-binding subunit ClpB | *Ehrlichia chaffeensis* | 291 | 323 | 9.00E-06 | 72% | -1 | 0 |
| 62916 | 317 | wAv62916 | porphobilinogen deaminase | *wBm* | 2 | 259 | 1.00E-05 | 39% | -2 | 4 |
| 64666 | 1482 | wAv6466.1 | hypothetical protein | *wOv* | 1064 | 1108 | 0.11 | 93% | 2 | 0 |
|  |  | wAv6466.2 |  |  | 1108 | 1170 | 0.11 | 59% | 1 | 0 |
| 67545 | 102 | wAv67545.1 | ATP-dependent DNA helicase RecG | *wAv* | 544 | 717 | 2.00E-10 | 44% | 1 | 2 |
|  |  | wAv67545.2 |  |  | 737 | 1069 | 2.00E-10 | 31% | 2 | 5 |

BLASTX based annotation of all *A. viteae* contigs containing *Wolbachia* homologs with a BLASTN e-value less than 1e-05. All hits to *Wolbachia* genes by BLASTX were recorded, regardless of e-value. Abbreviations are as follows: *Wolbachia* endosymbiont of *Culex quinquefasciatus,* *wCq*; *Wolbachia* endosymbiont of *Drosophila simulans*, *wRi*; *Wolbachia* endosymbiont of *Brugia malayi, wBm*; *Wolbachia* endosymbiont of *Drosophila willistoni*, *wDw*; *Wolbachia* endosymbiont of *Muscidifurax uniraptor*, *wMu*; *Wolbachia* endosymbiont of *Onchocerca volvulus*, *wOv*; *Wolbachia* endosymbiont of *Armadillium vulgare*, *wAv*. The average length of a sequence with homology to a *Wolbachia* protein was 123.8±73.5bp. The average percent ID to a *Wolbachia* protein was 62.3±13.8%. According to Student’s t-test, this is significantly lower than the average percent identity to a nematode protein, 79.1±15.6% (p-value=.0001). The Student’s t-test indicates that the average percent identity to a *Wolbachia* protein is also significantly lower than the percent identity of a sequence to a *Wolbachia* gene on the nucleotide level (p-value=8e-10).
