# Supplementary material for: Endosymbiont DNA in Endobacteria-Free Filarial Nematodes Indicates Ancient Horizontal Genetic Transfer
Source: PLoS One. 2010 Jun 9;5(6):e11029. doi: 10.1371/journal.pone.0011029 (PMC2882956; doi:10.1371/journal.pone.0011029)
Supplement: Table S4 — BLASTX annotation of O. flexuosa genomic DNA fragments. BLASTX based annotation of all O. flexuosa genomic fragmetns containing Wolbachia homologs with a BLASTN e-value better than 1e-05. All hits to Wolbachia genes by BLASTX were recorded, regardless of e-value. Abbreviations are as follows: Wolbachia endosymbiont of Drosophila simulans, wRi; Wolbachia endosymbiont of Onchocerca volvulus, wOv; Wolbachia endosymbiont of Drosophila melanogaster, wDm; Wolbachia endosymbiont of Brugia malayi, wBm; Wolbachia endosymbiont of Culex quinquefasciatus, wCq; Wolbachia endosymbiont of Dirofilaria immitis, wDi; Wolbachia endosymbiont of Drosophila willistoni, wDw; Wolbachia endosymbiont of Muscidifurax uniraptor, wMu. The average length of a sequence with homology to a Wolbachia protein was 148.3 plus or minus 121.2bp. The average percent ID to a Wolbachia protein was 67.3 plus or minus 13.2%. According to Student's t-test, this is significantly lower than the average percent identity to a nematode protein, 76.3 plus or minus 16.0% (p-value = .00014). The Student's t-test indicates that the average percent identity to a Wolbachia protein is also significantly lower than the percent identity of a sequence to a Wolbachia gene on the nucleotide level (p-value = 2.03e-15). (0.15 MB DOC) [file pone.0011029.s004.doc]

**Table S4.** BLASTX annotation of *O. flexuosa* genomic DNA fragments

| **Fragment #** | **Length (bp)** | **Locus Name** | **Best Annotation** | **Homolog Species** | **5' coord** | **3' coord** | **e value** | **%ID** | **Reading Frame** | **# stop codons** |
| --- | --- | --- | --- | --- | --- | --- | --- | --- | --- | --- |
| 1 | 1722 | wOf1 | Type IV secretion system protein VirB6 | *wRi* | 2 | 115 | 5.00E-06 | 63% | -3 | 0 |
|  |  | Of1.1 | hypothetical protein Bm1_25580 | *B. malayi* | 474 | 686 | 6.00E-23 | 88% | -2 | 0 |
|  |  | Of1.2 |  |  | 932 | 1198 | 9.00E-40 | 91% | -3 | 0 |
|  |  | Of1.3 |  |  | 1418 | 1534 | 4.00E-11 | 87% | -3 | 0 |
| 2 | 3502 | wOf2 | ferredoxin, iron-sulfur cluster assembly system | *wRi* | 3294 | 3494 | 9.2 | 44% | 3 | 5 |
| 3 | 2519 | wOf3.1 | lipoprotein releasing system transmembrane protein lolc | *wOv* | 1307 | 1402 | 1.00E-14 | 78% | -2 | 1 |
|  |  | wOf3.2 |  |  | 1450 | 1905 | 1.00E-14 | 38% | -3 | 9 |
|  |  | wOf3.3 |  |  | 1605 | 2381 | 2.00E-53 | 52% | -1 | 11 |
| 4 | 216 | wOf4.1 | delta-aminolevulinic acid dehydratase | *wDm* | 3 | 98 | 2.00E-05 | 86% | 3 | 0 |
|  |  | wOf4.2 |  |  | 107 | 202 | 2.00E-05 | 62% | 2 | 0 |
| 5 | 2453 | Of5.1 | cDNA sequence BC017158 | *B. malayi* | 116 | 451 | 4.00E-42 | 76% | -2 | 1 |
|  |  | Of5.2 |  |  | 900 | 1169 | 2.00E-28 | 71% | -1 | 0 |
|  |  | wOf5.1 | acid phosphatase | *wBm* | 2111 | 2284 | 3.00E-33 | 79% | -2 | 2 |
|  |  | wOf5.2 |  |  | 2287 | 2415 | 3.00E-33 | 81% | -3 | 1 |
| 6 | 172 | wOf6.1 | sodium/alanine symporter family protein | *wCq* | 7 | 69 | 3.00E-05 | 66% | 1 | 0 |
|  |  | wOf6.2 | sodium/alanine symporter family protein |  | 87 | 164 | 3.00E-05 | 77% | 3 | 0 |
| 7 | 3522 | Of7.1 | FERM domain | *B. malayi* | 3 | 818 | 7.00E-116 | 80% | -2 | 0 |
|  |  | Of7.2 | FERM domain |  | 782 | 922 | 4.00E-23 | 76% | -3 | 0 |
|  |  | Of7.3 | FERM domain |  | 1003 | 1092 | 4.00E-23 | 86% | -1 | 0 |
|  |  | Of7.4 | FERM domain |  | 1295 | 1465 | 2.00E-24 | 89% | -3 | 0 |
|  |  | Of7.5 | FERM domain |  | 2468 | 2554 | 7.00E-08 | 100% | -3 | 0 |
|  |  | Of7.6 | FERM domain |  | 2889 | 3119 | 1.00E-30 | 85% | -2 | 0 |
|  |  | wOf7 | Na+/alanine symporter | *wRi* | 3361 | 3450 | 1.00E-05 | 86% | -2 | 0 |
| 8 | 2511 | Of8 | Ser/Thr protein phosphatase family protein | *B. malayi* | 2224 | 2370 | 5.00E-09 | 57% | -1 | 0 |
| 9 | 3504 | Of9.1 | hypothetical protein | *B. malayi* | 932 | 1087 | 4.00E-12 | 71% | -3 | 0 |
|  |  | Of9.2 | hypothetical protein |  | 1378 | 1539 | 0.001 | 61% | -1 | 0 |
|  |  | wOf9 | type IV secretion system protein VirD4 | *wCq* | 3343 | 3429 | 8.00E-05 | 79% | 1 | 0 |
| 10 | 275 | wOf10 | aspartyl-tRNA synthetase | *wDm* | 30 | 233 | 9.00E-12 | 69% | 3 | 0 |
| 11 | 251 | wOf11 | guanylate kinase | *wRi* | 158 | 247 | 2.00E-05 | 80% | 2 | 0 |
| 13 | 4605 | Of13a.1 | Pao retrotransposon peptidase family protein | *B. malayi* | 777 | 938 | 3.00E-34 | 40% | 3 | 0 |
|  |  | Of13a.2 | Pao retrotransposon peptidase family protein |  | 935 | 1117 | 3.00E-34 | 34% | 2 | 1 |
|  |  | Of13a.3 | Pao retrotransposon peptidase family protein |  | 1168 | 1404 | 3.00E-34 | 56% | 1 | 0 |
|  |  | Of13b | carbamyl-phosphate synthase, large subunit (carB) | *Anaplasma marginale* | 1447 | 1578 | 3.00E-10 | 75% | 1 | 0 |
|  |  | wOf13 | putative cell division protein FtsK | *wCq* | 2055 | 2225 | 4.00E-14 | 71% | -2 | 1 |
|  |  | Of13c | Helix-loop-helix DNA-binding domain containing protein | *B. malayi* | 4054 | 4473 | 1.00E-41 | 59% | -1 | 0 |
| 14 | 251 | wOf14 | cytochrome b561, putative | *wDm* | 13 | 150 | 1.00E-08 | 62% | 1 | 0 |
| 15 | 2973 | Of15.1 | Myosin head containing protein | *B. malayi* | 679 | 861 | 2.00E-15 | 68% | -1 | 0 |
|  |  | Of15.2 | Myosin head containing protein |  | 1074 | 1211 | 4.00E-13 | 80% | -2 | 0 |
|  |  | Of15.3 | Myosin head containing protein |  | 1405 | 1548 | 6.00E-13 | 77% | -1 | 0 |
|  |  | wOf15 | valyl-tRNA synthetase | *wCq* | 2767 | 2973 | 3.00E-20 | 78% | 1 | 2 |
| 16 | 7725 | wOf16a | arginyl-tRNA synthetase | *wDi* | 6085 | 6213 | 1.00E-09 | 76% | -1 | 0 |
|  |  | wOf16b | topoisomerase IA, TopA | *wBm* | 6213 | 6329 | 9.00E-09 | 82% | -2 | 0 |
|  |  | wOf16c | rod shape-determining protein RodA | *wCq* | 6344 | 6514 | 7.00E-16 | 80% | 2 | 0 |
| 17 | 1123 |  | no hit |  |  |  |  |  |  |  |
| 18 | 164 | wOf18 | outer membrane protein/protective antigen OMA87 | *wBm* | 101 | 163 | 2.6 | 76% | -2 | 0 |
| 19 | 3075 | wOf19.1 | DNA mismatch repair protein MutL | *wCq* | 2855 | 3007 | 3.00E-08 | 60% | -3 | 1 |
|  |  | wOf19.2 | DNA mismatch repair protein MutL |  | 3024 | 3071 | 3.00E-08 | 75% | -2 | 0 |
| 20 | 193 | wOf20 | outer membrane protein | *wBm* | 9 | 179 | 6.00E-05 | 50% | -3 | 0 |
| 21 | 256 | wOf21.1 | triose-phosphate isomerase | *wDi* | 91 | 123 | 2.00E-05 | 72% | -2 | 0 |
|  |  | wOf21.2 | triose-phosphate isomerase |  | 122 | 208 | 2.00E-05 | 65% | -1 | 0 |
| 22 | 1955 | Of22.1 | Sm protein | *B. malayi* | 213 | 338 | 3.00E-08 | 71% | 3 | 0 |
|  |  | Of22.2 | Sm protein |  | 820 | 1044 | 2.00E-23 | 68% | 1 | 0 |
|  |  | wOf22.1 | type IV secretion system VirD4 protein | *wOv* | 1768 | 1806 | 4.00E-19 | 92% | 1 | 0 |
|  |  | wOf22.2 | type IV secretion system VirD4 protein |  | 1805 | 1954 | 4.00E-19 | 84% | 2 | 0 |
| 23 | 1299 | wOf23 | Glutamyl-tRNA amidotransferase subunit A | *wOv* | 64 | 162 | 0.01 | 66% | -1 | 0 |
| 24 | 257 | wOf24 | RNA polymerase sigma-32 factor | *wOv* | 133 | 234 | 0.14 | 61% | 1 | 1 |
| 25 | 258 | wOf25 | Putative oxidoreductase | *wCq* | 142 | 198 | 0.029 | 89% | 1 | 0 |
| 26 | 274 | wOf26 | ribonucleoside-diphosphate reductase alpha chain | *wOv* | 138 | 272 | 0.53 | 48% | -3 | 2 |
| 27 | 233 |  | no hit |  |  |  |  |  |  |  |
| 28 | 2859 | wOf28a | hypothetical protein WPa_0203 | *wCq* | 113 | 229 | 9.00E-09 | 79% | 2 | 0 |
|  |  | wOf28b | hypothetical protein | *wOv* | 456 | 743 | 2.00E-16 | 50% | 3 | 3 |
|  |  | Of28.1 | Histidine acid phosphatase family protein | *B. malayi* | 1316 | 1405 | 3.00E-08 | 90% | 2 | 0 |
|  |  | Of28.2 | Histidine acid phosphatase family protein |  | 1670 | 1819 | 9.00E-14 | 72% | 2 | 0 |
| 29 | 196 |  | no hit |  |  |  |  |  |  |  |
| 30 | 1923 | Of30 | metallo hydrolase | *Mycoplasma pneumoniae* | 403 | 549 | 2.00E-05 | 46% | 1 | 0 |
| 31 | 123 | wOf31 | DNA polymerase III, beta subunit | *wCq* | 52 | 123 | 7.00E-04 | 91% | 1 | 0 |
| 32 | 3316 | Of32 | PREDICTED: similar to hCG32758 | *Monodelphis domestica* | 1451 | 1765 | 3.00E-27 | 53% | 2 | 0 |
| 33 | 2269 | wOf33 | putative translation elongation factor G | *wDi* | 2191 | 2268 | 0.01 | 73% | -2 | 1 |
| 34 | 1772 | wOf34 | phosphopantetheine adenylyltransferase | *wBm* | 17 | 226 | 2.00E-06 | 48% | -2 | 4 |
| 35 | 5594 | Of35a.1 | Vacuolar h atpase protein 16 | *B. malayi* | 850 | 1491 | 3.00E-57 | 62% | -3 | 7 |
|  |  | Of35a.2 |  |  | 1730 | 1888 | 4.00E-21 | 98% | -2 | 0 |
|  |  | Of35a.3 |  |  | 2190 | 2444 | 1.00E-24 | 74% | -1 | 1 |
|  |  | Of35a.4 |  |  | 2446 | 2583 | 8.00E-22 | 73% | -3 | 0 |
|  |  | Of35a.5 |  |  | 2658 | 2744 | 8.00E-22 | 93% | -1 | 0 |
|  |  | Of35a.6 |  |  | 3053 | 3223 | 2.00E-13 | 70% | -2 | 0 |
|  |  | wOf35 | Peptidase S66 family protein | *wCq* | 4048 | 4125 | 0.013 | 69% | -1 | 0 |
|  |  | Of35b | Protein kinase domain containing protein | *B. malayi* | 5045 | 5287 | 1.00E-25 | 72% | 2 | 1 |
| 36 | 3218 | wOf36a | hypothetical protein WwSim0452 | *wRi* | 14 | 109 | 8.70E+00 | 62% | -2 | 0 |
|  |  | Of36.1 | hypothetical protein | *B. malayi* | 218 | 325 | 5.40E-01 | 66% | 2 | 0 |
|  |  | wOf36b.1 | ABC-type Fe3+ transport system, permease component | *wBm* | 526 | 609 | 8.10E+00 | 64% | -3 | 3 |
|  |  | wOf36b.2 |  |  | 633 | 668 | 8.10E+00 | 66% | -1 | 0 |
|  |  | Of36.2 | hypothetical protein | *B. malayi* | 1515 | 1638 | 2.00E-06 | 63% | 3 | 0 |
|  |  | Of36.3 |  |  | 2188 | 2340 | 6.00E-04 | 55% | 1 | 0 |
|  |  | Of36.4 |  |  | 2781 | 2975 | 3.00E-07 | 52% | 3 | 0 |
| 37 | 3335 | Of37a.1 | deoxyribose-phosphate aldolase | *B. malayi* | 598 | 720 | 5.00E-09 | 78% | 1 | 0 |
|  |  | Of37a.2 |  |  | 1097 | 1232 | 4.00E-10 | 75% | 2 | 0 |
|  |  | Of37b.1 | pyridine nucleotide-disulphide oxidoreductase family protein | *B. malayi* | 2207 | 2356 | 1.00E-15 | 88% | 2 | 0 |
|  |  | Of37b.2 |  |  | 2577 | 2693 | 3.00E-09 | 79% | 3 | 0 |
|  |  | Of37b.3 |  |  | 2918 | 2992 | 1.00E-21 | 88% | 2 | 0 |
|  |  | Of37b.4 |  |  | 3084 | 3209 | 1.00E-21 | 95% | 3 | 0 |
| 38 | 2580 | wOf38 | amidophosphoribosyltransferase | *wOv* | 65 | 271 | 4.00E-20 | 76% | -3 | 1 |
|  |  | Of38.1 | DAN domain containing protein | *B. malayi* | 309 | 452 | 8.00E-17 | 91% | 3 | 0 |
|  |  | Of38.2 |  |  | 713 | 853 | 3.00E-19 | 93% | 2 | 0 |
|  |  | Of38.3 |  |  | 1046 | 1165 | 1.00E-04 | 95% | 2 | 0 |
| 39 | 1172 |  | no hit |  |  |  |  |  |  |  |
| 40 | 190 |  | no hit |  |  |  |  |  |  |  |
| 41 | 84 |  | no hit |  |  |  |  |  |  |  |
| 42 | 201 | wOf42 | Short-chain alcohol dehydrogenase family enzyme | *wBm* | 1 | 144 | 3.00E-11 | 71% | -1 | 0 |
| 43 | 1889 | wOf43.1 | hydrolase, alpha/beta fold family | *wCq* | 31 | 138 | 1.00E-10 | 75% | -3 | 0 |
|  |  | wOf43.2 |  |  | 132 | 185 | 1.00E-10 | 77% | -1 | 0 |
|  |  | Of43 | EF hand family protein | *B. malayi* | 1231 | 1326 | 3.00E-05 | 71% | -3 | 0 |
| 44 | 2550 | Of44.1 | hypothetical protein SORBIDRAFT_06g016400 | *Sorghum bicolor* | 69 | 623 | 4.00E-30 | 33% | 3 | 6 |
|  |  | Of44.2 |  |  | 710 | 1159 | 4.00E-30 | 31% | 3 | 5 |
| 45 | 55 |  | no hit |  |  |  |  |  |  |  |
| 46 | 249 | wOf46 | 2-nitropropane dioxygenase-like protein | *wBm* | 13 | 177 | 5.00E-15 | 69% | -1 | 1 |
| 47 | 154 | wOf47 | heat shock protein 90 | *wRi* | 4 | 99 | 7.00E-06 | 78% | 1 | 0 |
| 48 | 234 | wOf48 | hypothetical protein WRi_002120 | *wRi* | 40 | 231 | 6.00E-05 | 73% | -1 | 0 |
| 49 | 3256 | wOf49 | FKBP-type peptidyl-prolyl cis-trans isomerase, TIG | *wBm* | 2944 | 3255 | 1.00E-19 | 84% | -2 | 1 |
| 50 | 285 | wOf50a | Type IV secretory pathway, VirB4 component | *wDw* | 34 | 144 | 1.00E-05 | 67% | 1 | 1 |
|  |  | wOf50b | hypothetical protein Wendoof_01000316 | *wDw* | 195 | 257 | 6.20E-02 | 71% | 3 | 0 |
| 51 | 3999 | Of51a | Dehydrogenases, short chain protein 30 | *B. malayi* | 3 | 326 | 5.00E-45 | 82% | 1 | 0 |
|  |  | Of51b.1 | ShTK domain containing protein | *B. malayi* | 2300 | 2398 | 8.00E-11 | 84% | -3 | 0 |
|  |  | Of51b.2 |  |  | 2594 | 3022 | 2.00E-24 | 44% | -3 | 0 |
|  |  | Of51b.3 |  |  | 3377 | 3484 | 2.00E-05 | 60% | -3 | 0 |
|  |  | wOf51 | N utilization substance protein A | *wCq* | 3836 | 3994 | 5.00E-07 | 57% | 2 | 1 |
| 52 | 286 | wOf52 | thioredoxin reductase | *wBm* | 1 | 165 | 3.00E-06 | 56% | -2 | 1 |
| 53 | 2411 | wOf53.1 | 2-methylthioadenine synthetase | *wBm* | 169 | 471 | 2.00E-42 | 82% | 1 | 0 |
|  |  | wOf53b.2 |  |  | 471 | 521 | 2.00E-42 | 82% | 3 | 0 |
|  |  | Of53.1 | signal peptide peptidase family protein | *B. malayi* | 1434 | 1628 | 2.00E-24 | 47% | -2 | 1 |
|  |  | Of53.2 |  |  | 1715 | 2125 | 2.00E-24 | 40% | -3 | 0 |
| 54 | 2749 | wOf54 | Type IV secretion system protein VirB4, putative | *wMu* | 2 | 130 | 5.00E-10 | 79% | -1 | 1 |
|  |  | Of54 | hypothetical protein Y73E7A.6 | *C. elegans* | 1599 | 1739 | 4.00E-10 | 63% | 3 | 0 |
| 55 | 263 | wOf55 | heme exporter protein CcmC | *wRi* | 2 | 289 | 4.00E-15 | 48% | 2 | 1 |
| 56 | 478 | wOf56 | hypothetical protein Wbm0633 | *wBm* | 177 | 281 | 3.00E-06 | 74% | -3 | 1 |
| 58 | 278 | wOf58.1 | transcription elongation factor NusA | *wBm* | 96 | 158 | 4.50E+00 | 71% | -1 | 0 |
|  |  | wOf58.2 |  |  | 183 | 278 | 3.00E-03 | 71% | 3 | 0 |
| 59 | 275 | wOf59 | amino acid permease family protein | *wCq* | 53 | 136 | 0.002 | 78% | -2 | 1 |
| 61 | 266 | wOf61 | amidophosphoribosyltransferase | *wOv* | 12 | 158 | 5.00E-04 | 65% | 3 | 1 |
| 62 | 232 | wOf62 | protoheme IX farnesyltransferase | *wBm* | 14 | 217 | 2.00E-13 | 85% | 2 | 0 |
| 63 | 254 |  | no hit |  |  |  |  |  |  |  |
| 64 | 259 | wOf64 | poly A polymerase family protein | *wMu* | 4 | 222 | 1.00E-09 | 53% | -2 | 2 |
| 65 | 301 | wOf65 | excinuclease ABC, C subunit | *wMu* | 146 | 208 | 1.2 | 71% | -1 | 0 |
| 66 | 257 |  | no hit |  |  |  |  |  |  |  |
| 67 | 253 | wOf67 | phosphatidylserine decarboxylase proenzyme | *wCq* | 3 | 65 | 2.70E+00 | 71% | -3 | 0 |
| 68 | 4116 | Of68a | histone H3 | *B. malayi* | 293 | 706 | 3.00E-69 | 97% | -3 | 0 |
|  |  | Of68b | linker histone H1 and H5 family protein |  | 1815 | 2189 | 2.00E-69 | 76% | -3 | 0 |
|  |  | wOf68a | ribosomal protein S11 | *wRi* | 3459 | 3581 | 1.00E-07 | 73% | -3 | 0 |
|  |  | wOf68b | GTP-binding protein TypA |  | 3716 | 4081 | 3.00E-08 | 36% | 3 | 3 |
| 69 | 5266 | Of69.1 | Egg laying defective protein 4, isoform c | *B. malayi* | 86 | 232 | 5.00E-17 | 89% | 2 | 0 |
|  |  | Of69.2 |  |  | 567 | 725 | 1.00E-18 | 84% | 3 | 1 |
|  |  | Of69.3 |  |  | 854 | 1396 | 3.00E-71 | 75% | 2 | 1 |
|  |  | Of69.4 |  |  | 1568 | 1762 | 8.00E-28 | 93% | 2 | 0 |
|  |  | Of69.5 |  |  | 1919 | 2080 | 3.00E-26 | 98% | 2 | 0 |
|  |  | wOf69 | TPR domain-containing protein | *wDi* | 5120 | 5266 | 1.00E-09 | 72% | -3 | 1 |
| 70 | 6909 | wOf70.1 | outer membrane protein TolC, putative | *wBm* | 3383 | 3472 | 6.00E-42 | 56% | -3 | 0 |
|  |  | wOf70.2 |  |  | 3496 | 4230 | 6.00E-42 | 42% | -1 | 6 |
|  |  | wOf70.3 |  |  | 4265 | 4342 | 6.00E-42 | 53% | -3 | 0 |
| 71 | 230 | wOf71.1 | Probable serine protease, HtrA | *wOv* | 58 | 165 | 3.00E-05 | 75% | 1 | 0 |
|  |  | wOf71.2 |  |  | 218 | 427 | 5.00E-28 | 63% | -2 | 0 |
|  |  | wOf71.3 |  |  | 424 | 546 | 5.00E-28 | 70% | -3 | 0 |
| 72 | 3684 | wOf72.1 | ATP-dependent Zn protease, HflB | *wBm* | 87 | 254 | 5.00E-57 | 61% | -2 | 3 |
|  |  | Wof72.2 |  |  | 256 | 591 | 5.00E-57 | 81% | -1 | 1 |
|  |  | Of72.1 | Actin family protein | *B. malayi* | 1404 | 1853 | 1.00E-28 | 50% | 3 | 2 |
|  |  | Of72.2 |  |  | 2176 | 2403 | 3.00E-29 | 86% | 1 | 0 |
|  |  | Of72.3 |  |  | 2633 | 2755 | 4.00E-07 | 93% | 2 | 0 |
|  |  | Of72.4 |  |  | 3023 | 3175 | 6.00E-07 | 88% | 2 | 0 |
|  |  | Of72.5 |  |  | 3376 | 3639 | 4.00E-20 | 60% | 1 | 1 |
| 73 | 255 | wOf73.1 | 3-octaprenyl-4-hydroxybenzoate carboxy-lyase | *wDm* | 15 | 92 | 4.80E-02 | 59% | -2 | 1 |
|  |  | wOf73.2 |  |  | 145 | 255 | 4.80E-02 | 47% | -1 | 2 |
| 74 | 184 |  | no hit |  |  |  |  |  |  |  |
| 75 | 203 |  | no hit |  |  |  |  |  |  |  |
| 76 | 2675 | wOf76.1 | ABC-type phosphate transport system | *wDi* | 34 | 216 | 8.00E-27 | 95% | -3 | 0 |
|  |  | wOf76.2 |  |  | 195 | 257 | 8.00E-27 | 57% | -1 | 0 |
|  |  | Of76 | hypothetical protein SORBIDRAFT_09g016160 | *Sorghum bicolor* | 415 | 1269 | 1.00E-11 | 27% | -3 | 9 |
| 77 | 278 |  | no hit |  |  |  |  |  |  |  |
| 78 | 2506 |  | no hit |  |  |  |  |  |  |  |
| 79 | 269 |  | no hit |  |  |  |  |  |  |  |
| 80 | 1184 |  | no hit |  |  |  |  |  |  |  |
| 81 | 263 | wOf81 | dihydroorotase | *wCq* | 151 | 261 | 0.004 | 67% | -3 | 1 |
| 82 | 271 |  | no hit |  |  |  |  |  |  |  |
| 83 | 224 | wOf83 | DNA mismatch repair protein MutL | *wCq* | 44 | 121 | 3.5 | 61% | 2 | 0 |
| 84 | 158 | wOf84 | inner membrane protein translocase component YidC | *wBm* | 27 | 143 | 2 | 56% | 3 | 0 |
| 85 | 235 |  | no hit |  |  |  |  |  |  |  |
| 86 | 2775 | Of86.1 | hypothetical protein Bm1_29410 | *B. malayi* | 292 | 444 | 4.00E-24 | 100% | -1 | 0 |
|  |  | Of86.2 |  |  | 683 | 793 | 2.00E-10 | 97% | -3 | 0 |
|  |  | Of86.3 |  |  | 2332 | 2514 | 5.00E-14 | 86% | -1 | 0 |
| 87 | 150 |  | no hit |  |  |  |  |  |  |  |
| 88 | 2786 | wOf88 | DNA polymerase I | *wRi* | 99 | 488 | 2.00E-17 | 49% | -1 | 6 |
|  |  | Of88.1 | hypothetical protein | *B. malayi* | 573 | 992 | 3.00E-48 | 67% | -1 | 0 |
|  |  | Of88.2 |  |  | 1078 | 1179 | 3.00E-48 | 94% | -3 | 0 |
|  |  | Of88.3 |  |  | 1560 | 1733 | 1.00E-21 | 85% | -1 | 0 |
|  |  | Of88.4 |  |  | 1929 | 2054 | 9.00E-13 | 83% | -1 | 0 |
|  |  | Of88.5 |  |  | 2291 | 2572 | 5.00E-40 | 85% | -2 | 0 |
| 89 | 3175 | wOf89.1 | ATP synthase F1, delta subunit | *wCq* | 2888 | 2980 | 0.01 | 51% | -1 | 0 |
|  |  | wOf98.2 |  |  | 3076 | 3144 | 0.01 | 65% | -2 | 0 |
|  |  | wOf98.3 |  |  | 3117 | 3173 | 0.01 | 47% | -3 | 1 |
| 90 | 193 |  | no hit |  |  |  |  |  |  |  |
| 91 | 264 |  | no hit |  |  |  |  |  |  |  |
| 92 | 258 |  | no hit |  |  |  |  |  |  |  |
| 93 | 264 |  | no hit |  |  |  |  |  |  |  |
| 94 | 2662 | wOf94.1 | SPFH domain/Band 7 family protein | *wMu* | 45 | 122 | 1.00E-04 | 53% | 3 | 1 |
|  |  | wOf94.2 |  |  | 124 | 312 | 1.00E-04 | 42% | 1 | 4 |
| 95 | 1385 | wOf95a.1 | ribonuclease H | *wCq* | 183 | 248 | 9.00E-14 | 63% | -1 | 0 |
|  |  | wOf95a.2 | ribonuclease H |  | 251 | 397 | 9.00E-14 | 61% | -2 | 1 |
|  |  | wOf95b | putative monovalent cation/H+ antiporter subunit D | *wDw* | 366 | 614 | 6.00E-14 | 54% | -1 | 2 |

BLASTX based annotation of all *O. flexuosa* genomic fragmetns containing *Wolbachia* homologs with a BLASTN e-value better than 1e-05. All hits to *Wolbachia* genes by BLASTX were recorded, regardless of e-value. Abbreviations are as follows: *Wolbachia* endosymbiont of *Drosophila simulans*, *wRi*; *Wolbachia* endosymbiont of *Onchocerca volvulus*, *wOv*; *Wolbachia* endosymbiont of *Drosophila melanogaster, wDm*; *Wolbachia* endosymbiont of *Brugia malayi, wBm*; *Wolbachia* endosymbiont of *Culex quinquefasciatus,* *wCq*; *Wolbachia* endosymbiont of *Dirofilaria immitis*, *wDi*; *Wolbachia* endosymbiont of *Drosophila willistoni*, *wDw*; *Wolbachia* endosymbiont of *Muscidifurax uniraptor*, *wMu*. The average length of a sequence with homology to a *Wolbachia* protein was 148.3±121.2bp. The average percent ID to a *Wolbachia* protein was 67.3±13.2%. According to Student’s t-test, this is significantly lower than the average percent identity to a nematode protein, 76.3±16.0% (p-value=.00014). The Student’s t-test indicates that the average percent identity to a *Wolbachia* protein is also significantly lower than the percent identity of a sequence to a *Wolbachia* gene on the nucleotide level (p-value=2.03e-15).
