# Supplementary material for: Endosymbiont DNA in Endobacteria-Free Filarial Nematodes Indicates Ancient Horizontal Genetic Transfer
Source: PLoS One. 2010 Jun 9;5(6):e11029. doi: 10.1371/journal.pone.0011029 (PMC2882956; doi:10.1371/journal.pone.0011029)
Supplement: Table S5 — Results of qPCR expression studies and presence of potential open reading frames. Table describes the results of qRT-PCR studies used to determine whether Wolbachia homologs described in Tables S1 and S2 are expressed at the transcript level. + indicates that the sequenced is expressed at the RNA level while - indicates that it was not. n/a indicates that the sequence could not be tested using this method (for example, the sequence was too short or AT rich to design qRT-PCR primers). Sequences more than 25bp from the end of a genomic fragment are considered internal and sequences lacking premature stop codons and frameshift mutations are reported as potential open reading frames. Defined start and stop codons were not required for classification as a potential open reading frame. (0.15 MB DOC) [file pone.0011029.s005.doc]

**Table S5.** Results of qPCR expression studies and presence of potential open reading frames.

| Locus Name | Transcribed | Internal or End | BLASTN length | Potential ORF | notes |
| --- | --- | --- | --- | --- | --- |
| wAv187 | + | internal | 137 | yes |  |
| wAv2452a | + | end | 129 | no | no predicted ORF or BLASTX hit |
| wAv2452b | n/a | internal | 69 | yes | predicted ORF, no BLASTX hit |
| wAv3046 | + | internal | 132 | yes | 2 small orfs/exons |
| wAv4552.1 | + | end | 177 | yes | predicted ORF, no BLASTX hit |
| wAv4766 | - | internal | 108 | yes |  |
| wAv5106a | + | end | 215 | no | no predicted ORF or BLASTX hit |
| wAv9153 | + | end | 306 | no | premature stops by BLASTX |
| wAv9497 | + | end | 195 | No | premature stops by BLASTX |
| wAv9524a | - | internal | 156 | No | premature stops by BLASTX |
| wAv9524b | - | internal | 170 | No | premature stops and frameshifts by BLASTX |
| wAv10096.1 | + | internal | 436 | No | premature stops by BLASTX |
| wAv1068 | n/a | internal | 116 | No | premature stops by BLASTX |
| wAv11045 | + | internal | 115 | maybe | overlaps predicited ORF |
| wAv11910 | - | end | 425 | No | premature stops by BLASTX |
| wAv13336a | + | internal | 135 | No | no predicted ORF or BLASTX hit |
| wAv13336b | + | internal | 98 | No | no predicted ORF or BLASTX hit |
| wAv16332 | + | internal | 312 | No | premature stops by BLASTX |
| wAv16679 | - | internal | 198 | Yes | predicted ORF, no BLASTX hit |
| wAv16952 | + | end | 162 | No | premature stops by BLASTX |
| wAv19080 | - | internal | 101 | Yes |  |
| wAv20032 | + | end | 130 | maybe | overlaps predicited ORF |
| wAv21852 | + | end | 174 | Yes |  |
| wAv22549 | + | end | 109 | No | no predicted ORF or BLASTX hit |
| wAv22942 | n/a | end | 59 | Yes | predicted ORF, no BLASTX hit |
| wAv24238 | - | internal | 182 | Yes | 2 small orfs/exons |
| wAv24716 | + | internal | 125 | Yes |  |
| wAv25554 | n/a | end | 188 | Yes | predicted ORF, no BLASTX hit |
| wAv28511 | + | end | 121 | Yes | overlaps predicited ORF |
| wAv29055 | + | end | 214 | Yes | 2 smal orfs/exons |
| wAv30005 | + | internal | 142 | Yes | 2 small orfs/exons |
| wAv30574a | - | internal | 59 | No | no predicted ORF or BLASTX hit |
| wAv30574b | n/a | internal | 82 | Yes | overlaps predicited ORF |
| wAv31107 | + | internal | 122 | Yes |  |
| wAv31515 | n/a | end | 126 | No | premature stops by BLASTX |
| wAv31988 | + | end | 158 | maybe | overlaps multiple small orfs |
| wAv35539 | - | end | 127 | No | no predicted ORF or BLASTX hit |
| wAv36441 | + | end | 114 | maybe | partially overalps predicted ORF |
| wAv38375 | + | internal | 56 | maybe | partially overalps predicted ORF |
| wAv38543 | + | internal | 223 | No | premature stops by BLASTX |
| wAv41791 | - | end | 128 | No | premature stops by BLASTX |
| wAv42190 | + | end | 177 | maybe | BLASTX only encompasses part of BLASTN |
| wAv46345 | + | end | 246 | maybe | BLASTX only encompasses part of BLASTN |
| wAv48068 | + | end | 104 | No | no predicted ORF or BLASTX hit |
| wAv52396 | n/a | internal | 113 | No | no predicted ORF or BLASTX hit |
| wAv55693 | + | internal | 144 | No | frameshift |
| wAv62916 | - | end | 216 | No | premature stops by BLASTX |
| wAv64666 | + | internal | 93 | No | frameshift |
| wAv67545 | - | internal | 295 | No | premature stops by BLASTX |
| wOf1a | + | end | 80 | Yes |  |
| wOf1b | + | internal | 128 | No | no predicted ORF or BLASTX hit |
| wOf2 | + | end | 107 | No | premature stops by BLASTX |
| wOf3 | n/a | end | 1214 | No | premature stops and frameshifts by BLASTX |
| wOf4 | + | end | 183 | Yes | 2 small orfs/exons |
| wOf5a | + | internal | 182 | maybe | partially overalps predicted ORF |
| wOf5b | + | internal | 320 | No | premature stops and frameshifts by BLASTX |
| wOf6 | n/a | end | 146 | No | premature stops and frameshifts by BLASTX |
| wOf7 | - | end | 166 | Yes |  |
| wOf8 | - | end | 102 | Yes |  |
| wOf9 | na/ | internal | 91 | Yes |  |
| wOf10 | + | end | 245 | Yes |  |
| wOf11 | + | end | 125 | Yes |  |
| wOf13a | na/ | internal | 181 | maybe | partially overalps predicted ORF |
| wOf13b | - | internal | 206 | No | no predicted ORF or BLASTX hit |
| wOf13c.1 | + | internal | 79 | No | no predicted ORF or BLASTX hit |
| wOf13c.2 | n/a | internal | 216 | No | no predicted ORF or BLASTX hit |
| wOf13d | - | internal | 98 | No | premature stops by BLASTX |
| wOf14 | + | internal | 78 | Yes |  |
| wOf15 | + | end | 202 | No | premature stops by BLASTX |
| wOf16a | - | internal | 100 | Yes | predicted ORF, no BLASTX hit |
| wOf16b | - | internal | 125 | Yes |  |
| wOf16c | + | internal | 124 | Yes |  |
| wOf16d | - | internal | 164 | Yes |  |
| wOf16e | n/a | internal | 61 | No | no predicted ORF or BLASTX hit |
| wOf19.1 | + | internal | 107 | No | premature stops by BLASTX |
| wOf19.2 | n/a | end | 75 | Yes |  |
| wOf20 | + | internal | 86 | Yes |  |
| wOf21 | - | internal | 169 | No | frameshift |
| wOf22 | + | end | 185 | No | frameshift |
| wOf23 | + | internal | 155 | Yes |  |
| wOf24 | + | internal | 117 | No | premature stops by BLASTX |
| wOf25 | n/a | internal | 57 | Yes |  |
| wOf26.1 | - | end | 159 | No | only partially overlaps BLASTX |
| wOf26.1 | n/a | end | 67 | No | premature stops by BLASTX |
| wOf27 | n/a | end | 59 | Yes | predicted ORF, no BLASTX hit |
| wOf28a | + | internal | 141 | Yes |  |
| wOf28b | + | internal | 484 | No | premature stops by BLASTX |
| wOf28c | - | internal | 117 | No | no predicted ORF or BLASTX hit |
| wOf29 | - | end | 136 | No | no predicted ORF or BLASTX hit |
| wOf30 | n/a | end | 45 | maybe | predicted ORF, no BLASTX hit |
| wOf31 | n/a | end | 71 | Yes |  |
| wOf32 | + | internal | 118 | No | no predicted ORF or BLASTX hit |
| wOf33 | n/a | end | 72 | No | premature stops by BLASTX |
| wOf34 | - | internal | 123 | No | premature stops by BLASTX |
| wOf35 | + | internal | 178 | maybe | only partially overlaps BLASTX |
| wOf36.1 | + | internal | 58 | Yes |  |
| wOf36.2 | + | internal | 64 | yes | predicted ORF (both exons), no BLASTX hit |
| wOf36b | n/a | internal | 140 | no | premature stops by BLASTX |
| wOf37 | + | end | 77 | no | no predicted ORF or BLASTX hit |
| wOf38 | n/a | internal | 201 | no | premature stops by BLASTX |
| wOf39 | n/a | end | 53 | no | no predicted ORF or BLASTX hit |
| wOf40 | - | end | 148 | maybe | partially overalps predicted ORF |
| wOf41 | n/a | end | 46 | maybe | partially overalps predicted ORF |
| wOf42a | + | end | 111 | yes |  |
| wOf42b | n/a | end | 78 | no | no predicted ORF or BLASTX hit |
| wOf43 | - | internal | 154 | yes | 2 short exons/ORFS |
| wOf44 | n/a | internal | 30 | yes | predicted ORF, no BLASTX hit |
| wOf45 | n/a | end | 53 | yes | predicted ORF, no BLASTX hit |
| wOf46 | + | internal | 128 | no | premature stops by BLASTX |
| wOf47 | + | end | 137 | yes |  |
| wOf48 | - | end | 190 | yes |  |
| wOf49a | - | internal | 164 | yes | predicted ORF, no BLASTX hit |
| wOf49b | + | internal | 101 | no | same exon as 49c, premature stops by BLASTX |
| wOf49c | n/a | end | 163 | no | same exon as 49b, premature stops by BLASTX |
| wOf50 | + | end | 221 | no | premature stops by BLASTX |
| wOf51 | + | end | 132 | no | premature stops by BLASTX |
| wOf52 | - | end | 188 | no | premature stops by BLASTX |
| wOf53a | - | end | 97 | yes | predicted ORF, no BLASTX hit |
| wOf53b | - | internal | 340 | no | frameshift |
| wOf53c | + | internal | 110 | yes | predicted ORF, no BLASTX hit |
| wOf53d | + | internal | 123 | no | no predicted ORF or BLASTX hit |
| wOf54 | - | end | 131 | no | premature stops by BLASTX |
| wOf55 | + | end | 289 | no | premature stops by BLASTX |
| wOf56 | n/a | internal | 137 | no | premature stops by BLASTX |
| wOf58.1 | n/a | end | 61 | yes | partially overalps predicted ORF |
| wOf58.2 | - | end | 104 | yes |  |
| wOf59 | + | internal | 141 | no | premature stops by BLASTX |
| wOf61 | + | end | 236 | no | premature stops by BLASTX |
| wOf62 | - | end | 224 | yes |  |
| wOf63 | + | end | 244 | maybe | partially overalps predicted ORF |
| wOf64 | + | end | 187 | no | premature stops by BLASTX |
| wOf65 | + | end | 162 | maybe | only partially overlaps BLASTX |
| wOf66 | + | end | 181 | no | no predicted ORF or BLASTX hit |
| wOf67 | n/a | end | 141 | maybe | only partially overlaps BLASTX |
| wOf68a | + | internal | 64 | no | no predicted ORF or BLASTX hit |
| wOf68b | + | internal | 392 | no | no predicted ORF or BLASTX hit |
| wOf68c | + | end | 513 | no | premature stops by BLASTX |
| wOf69 | + | end | 145 | no | premature stops by BLASTX |
| wOf70 | + | internal | 1672 | no | premature stops and frameshifts by BLASTX |
| wOf71.1 | + | internal | 117 | yes |  |
| wOf71.2 | n/a | end | 349 | no | frameshift |
| wOf72 | - | internal | 502 | no | premature stops and frameshifts by BLASTX |
| wOf73 | + | end | 162 | no | premature stops and frameshifts by BLASTX |
| wOf74 | + | end | 134 | no | no predicted ORF or BLASTX hit |
| wOf75 | - | end | 71 | yes | predicted ORF, no BLASTX hit |
| wOf76a | + | internal | 229 | yes | 2 small orfs/exons |
| wOf76b | - | end | 226 | no | no predicted ORF or BLASTX hit |
| wOf77 | + | internal | 166 | no | no predicted ORF or BLASTX hit |
| wOf78 | - | end | 160 | no | no predicted ORF or BLASTX hit |
| wOf79 | - | internal | 169 | no | no predicted ORF or BLASTX hit |
| wOf80 | + | internal | 145 | no | no predicted ORF or BLASTX hit |
| wOf81 | n/a | end | 109 | no | premature stops by BLASTX |
| wOf82 | + | internal | 95 | no | no predicted ORF or BLASTX hit |
| wOf83 | + | end | 100 | yes |  |
| wOf84 | - | end | 120 | yes |  |
| wOf85 | n/a | end | 81 | no | no predicted ORF or BLASTX hit |
| wOf86 | + | end | 95 | maybe | partially overalps predicted ORF |
| wOf87 | - | end | 103 | no | no predicted ORF or BLASTX hit |
| wOf88 | n/a | end | 484 | no | premature stops by BLASTX |
| wOf89.1 | n/a | internal | 185 | maybe | only partially overlaps BLASTX |
| wOf89.2 | n/a | end | 140 | yes |  |
| wOf90 | n/a | internal | 82 | yes | predicted ORF, no BLASTX hit |
| wOf91 | n/a | end | 117 | maybe | partially overalps predicted ORF |
| wOf92 | - | internal | 108 | no | no predicted ORF or BLASTX hit |
| wOf93 | n/a | internal | 74 | no | no predicted ORF or BLASTX hit |
| wOf94 | + | internal | 243 | no | premature stops and frameshifts by BLASTX |
| wOf95a | + | internal | 323 | no | premature stops and frameshifts by BLASTX |
| wOf95b | + | internal | 238 | no | premature stops by BLASTX |

Table describes the results of qRT-PCR studies used to determine whether *Wolbachia* homologs described in Tables S1 and S2 are expressed at the transcript level. + indicates that the sequenced is expressed at the RNA level while – indicates that it was not. n/a indicates that the sequence could not be tested using this method (for example, the sequence was too short or AT rich to design qRT-PCR primers). Sequences more than 25bp from the end of a genomic fragment are considered internal and sequences lacking premature stop codons and frameshift mutations are reported as potential open reading frames. Defined start and stop codons were not required for classification as a potential open reading frame.
