# Supplementary material for: Endosymbiont DNA in Endobacteria-Free Filarial Nematodes Indicates Ancient Horizontal Genetic Transfer
Source: PLoS One. 2010 Jun 9;5(6):e11029. doi: 10.1371/journal.pone.0011029 (PMC2882956; doi:10.1371/journal.pone.0011029)
Supplement: Table S6 — SYBR green qRT-PCR and RNA in situ hybridization probe primers. (0.14 MB DOC) [file pone.0011029.s006.doc]

**Table S6.** SYBR green qRT-PCR and RNA *in situ* hybridization probe primers.

| **Target** | **Forward Primer (5’-3’)** | **Reverse Primer (5’-3’)** |
| --- | --- | --- |
| wAv187 | CATTACAGGATTGCTTGTAGTGGCATA | CACGTGCAACATGATGAAAAGATATTG |
| wAv2452a | GAATTGAACTTCCGGCCTCA | GAACTTTTATTGATATTTTGCCTTGAA |
| wAv3046 | CGAGGCTTGACAATGCAGAG | ACTTGGCACCTCCAAGCAAG |
| wAv4552 | CCTGCATATATCGTTTGAGAATTCCATT | TGGCTCTTTTAGTATGGTTGATGAAGG |
| wAv4766 | AAAATGACAAAGGTATTTATAAAATGGTGGA | CTTAGAACGGCATATATCAAGATCATCC |
| wAv5106a | CATCAATTAACAGAAAGCGAATGG | CATTACACTTCATCTTCAAGTTTTCCT |
| wAv9153 | CAGCCAGGTTTGCAATCTACCA | CAACTGTTACGGCAGAATAACCAA |
| wAv9497 | GGTGTGCGGGAATTTCGTTG | TGTTGCGTGAGAAAGTCACTGC |
| wAv9524a | CAACATACAGCGCCATGAGG | AACAGGGAACGACAAAGACCA |
| wAv9524b | TTTCGGTCGAAACTATTGTGAAC | CAAATTTATTATAAGTCGCCGCAGA |
| wAv10096 | GCTTTGGCTGAATTTTTGCTTC | CCTTCGAGTTCTTCCCATAACAA |
| wAv10682 | CACTTGTACTAAAAATCAGTGTTCCACCA | CCATTCACAGCAAGGAGTGGTTT |
| wAv11045 | AATTTCTATTGATAATATGTTGATTGTGCTCTTG | TGCTGCTGTTTTATTTACGGTTGG |
| wAv11910 | ACAGAAAGTGCGGGTGCAAA | CATGGGACCCTCAGGTTTGG |
| wAv13336a | GGGAGAATTGAAAAAGTTGAGCAAGA | CTTTGTGTGTGCCGCTTTTGA |
| wAv13336b | TTTTGAAGGCATTAAAATGTGCTAATTG | TTTCATCAGCTGTAGTTGCTTTTCCA |
| wAv16332 | TCGCAATCCTTTCTGCATTG | TGAACAGCTTAGCGAAAAGCAC |
| wAv16679 | TGTGGATAGGGGACAAACGAA | TGCACCCCCTACCCTCTACA |
| wAv16952 | CGTTCTTGTGAAGTCGTATTGGTTG | CATCCGCATGAGGCTTAGAACC |
| wAv19080 | TTGTGGATCATAATTTGTTATCACTAAACG | CGAATATTTTTGATAATCTATTTCCTTATTGACG |
| wAv20032 | TCAATAGAGCTAATAACATGTTCCAATTT | TTGCAACTGGTACCCCTCATCTTAT |
| wAv21852 | ACTTTTCGAGAAACGACTCAGCA | CATCTAAAACTCCAGGCCATCC |
| wAv22549 | GCAAAGTTTGTATAAAATGACGGAAT | GCTGTAAACAACCGCTTAGCC |
| wAv24238 | CTGGCATTGCGCTAGCAGTT | TTCTGGCAGCTGCAAAATGTG |
| wAv24716 | GAAACACGTCAGTCAGCGTTTCA | GAACGATCCCGACCGAAGTC |
| wAv28511 | CCCAAAGAGAGCTCATTTCTCC | AAATTTGATGGTATTGACTGATTTGC |
| wAv29055 | TTTTTCAAATTTACCCTTTGATACACAA | TCAGCTGTGGGGAAAGCAAA |
| wAv30005 | TTCATTTAACGTACCTCCAGAGAATTT | GCGTTTGCTCTATAATTTTCTTATCTG |
| wAv30574a | TTCGTGGATTTTATGAGCTATTTGG | CAACAATACCAGAAATAATGTGTTCTGAC |
| wAv31107 | CCCATAAAGTTTCCGGATCTTACA | TCTCAATCAGCAGATTCCTTACG |
| wAv31515 | AAAAACTGGATTGCAGCATCA | TGCGCGCAAATAATGTGTTC |
| wAv31988 | CAGATCGCAGCTTCTTTTCAA | GCAATAAAATAATCCGGCGAAA |
| wAv35539 | TTGGTACTTTTGTAAAAGGAGAAAACA | AAATCTTAAGTTTCGTATATTATTTATCAC |
| wAv36441 | AGCCCTTAACCCACTTCTAA | GCTCTGCTTCAATCACCCATA |
| wAv38375 | TTACACCTTTTCCTCTCGAA | CGCGTGAAACGTGCATAACT |
| wAv41791 | TCTGTTTATTTTTCACTCTTTATGCTG | TTTTGCCGAGCAAGTGATGT |
| wAv42190 | CTGAGCATCGGCTGCAATAC | TTTTCCGTAATTGTACGACGAGAA |
| wAv46345 | CGCATTACACGTTCCACGTT | TTTTGCAATTTTGGCTCTTTAGC |
| wAv48068 | TGCATTTTGATAGTATTTCTTCTCAAAGG | CAGTTGTTAGTTATAAATGGTATATGTCG |
| wAv52396 | CACCCTTGAAACGAAAAAGG | CCGTTTCCTTCCTTGTCAAA |
| wAv55693 | TCGCATCACGCAAATCAAAT | ATTGGGTGATTCTGCTATTGGAA |
| wAv62916 | GCCTGTTGTGGTAAAGCCTCA | TCATTCGGTAAAGATGTCTCTCCA |
| wAv64666 | TGCTCAGGTGCTTCCTGAAA | GGCCCAAGACTAGCTGCAAA |
| wAv67546 | GGGGGAGAACAATTCGTATG | TCCTCGAGGCTGTGAATATAA |
| wOf1a | CGTATGACAAGAAATTTATGTTAAGCAA | GTTTTATTACAACCTCACAGACTTTTGGA |
| wOf1b | CGGCTTAGAAAATCAGCCAGCTA | TTGCTGTTTGATGAGCAGTCCA |
| wOf2 | AATTCTGTAGAAATACATAATCCCATATTTTGA | GCATCCAAGCCTCGATGTCTA |
| wOf4 | CAGCAAATGATAAGTTTAATTTCCCGATTTTT | GTTTGTTGCAACTTTTATCACTACACATTCA |
| wOf5a | GCTCCGCTATTATGTCTTATAGTACCATTG | CCAAACTGATCAAACTTCAAAGAAAA |
| wOf5b | TGGCGCAAAAACCTTTGTATTG | TGGTACTGTAATGGAAAGCACTGGA |
| wOf7 | CCTTCAAGGCCAAACCCTATATCTG | TATTCGAGTAGGAACTTTACGGGATATTTT |
| wOf8 | TCAGCATCAAGTGAGTTATTCTCAACA | CAATAAGGCAGTTGGCCTGAAA |
| wOf10 | TGCATTATCACAAGCTCCACAGA | CGGAAACATGGTGCAGTTTGA |
| wOf11 | TGAGCACTGGAGTGTTTTGCTT | GAAGGAGGCATAAAAATGCTTACAA |
| wOf13b | GCACAAAGCTCAAGGAGGTAATCC | GTGGGATCGGTGGCTGAAGT |
| wOf13c | TCGCTTACAAATGGCCGTGA | GGGGCTAAACAATTGCTCGGTA |
| wOf13d | TCATTTGTGAAAAGCGTGGAATTT | ACATATTCAACAGCAGCGGAACC |
| wOf14 | TCTTTATGCCAGAAGCAGAGGACA | TCTCGATTTGAAGCCAATTTAAGCA |
| wOf15 | CCGAAAGCAATTGACCGAAT | TCTCACGAGTTTTAACATCTGTACGAA |
| wOf16a | TGCCATAACAATCGGCGTTTC | TGCTGGGCTTAGATCTTTCAGAGTG |
| wOf16b | CAAACATCCCCACGTGCTTT | AAGCAAAGAAGTATCCTAAATACTAGTGCAGA |
| wOf16c | TCTGCACACCGCTGAAAGAAA | GGTAACAAAAGATTGATTCCGAGCA |
| wOf16d | TGCTCGCTATTTTAGTCAGCAAAGTG | TGGTTGTTTTAATACCAAGAACATAGACA |
| wOf19 | GCGGGAAATTCTCTTGCTTTGA | AAAATGGAATGGAAATGATTGAGATTTAT |
| wOf20 | GCTCTACAACCCACCCGAGTTT | TTTTACTGCTGCCTTAACTGGTGA |
| wOf21 | CGCCATATATCAGCAAGCGGTTT | AAAGATGCAGAAAGCTGTAAAAATAGGA |
| wOf22 | GCTTTTAATGCAAGTTTTCTATCACC | CACCATAAGGCTCGTCATCCA |
| wOf23 | GTGTTTGCGAAGCCCAAAGC | TCGGAATAGGCATGCTCGGTA |
| wOf24 | CGGAGTCATGAAGAAAAAGAATTAAAAGA | CAAAATGTCTTTTGACCTTCCGCTAA |
| wOf26 | TGACGAGCGAAAGAGGAAAAGG | CAAAACGTTCTGCTTATACAAGACTATCACC |
| wOf28a | AACATTGCCATCAGCAGCAA | TGGCAGTTTTGCTCGGTTAGA |
| wOf28b | TGGGGGCTCAACCTGATGAT | TCGCATTTCCGCTTTTCTAACC |
| wOf28c | TTCAAAATCAGTTCTTTTCAACAGACG | CTTGGAGAGATCTTCTCTTATTCAAGAGTTT |
| wOf29 | TTTCATTTCTATAAAGTTAGGGGAGCATTT | GGGTAACCACAAGATTTGGCAAT |
| wOf32 | ATTGCAAGAAAACATGGACTTGAAAA | TTACTCCAAACGTTTTCTTTAATACCCATC |
| wOf34 | TCGTGATACGATTGACGCTAATGTG | CCAAAGCTTTTCAGGCTATCAAAGT |
| wOf35 | CGTCCAGAACTAATTCGTGTCGAT | TGATTGGCAAACTTTTCGTAGCAC |
| wOf36a | CCAAATGTTGGCAAATCAGCA | GACTTCTTTCTCGCTCGGCTTA |
| wOf37 | GCTTGACCGAATCAGAGAAGGA | CCCCGCTTGTATTTCTATCTAGCC |
| wOf42a | GCTGCAAGTAAAGCTGCACTAGAAATC | GGCACTATGCTAATTTCTGTTTATTTTTACTTCA |
| wOf43 | CGGTCTGAAATAAAATTCCCGATG | TGATGTTGAAGTATATTTAACGAAATCATCAGA |
| wOf46 | TTTCCCCGAGCCTGTAAGACA | TCATGGCGTATTAGAAAAAGCAAAA |
| wOf47 | TTCATGAGGGTGCTATGGATCTT | CAACCACCTTAGGCGTGCAA |
| wOf48 | TTCATCAATTTTTCGCCATAAGC | GAAAACTGGAATTAAGATGTACGAAGCA |
| wOf49a | AAATGAAATTCGGCGCCTCAC | TTGTTGGTCTCTCAGCCAAAACG |
| wOf49b | GCTTTTCTGATTTTTCCGAGTCTGG | GCACAAAGTTATTGCGCAGCTT |
| wOf50 | AAGGGAAAAAGGCGACGAAG | CCTTGATACAAAAGACCAAATATCAGGA |
| wOf51 | AGTCTCAGAGCTTGTTGGATGGA | TCGGCCAATAAAACTGAACACTGA |
| wOf52 | TCCGACTGCATATATCCTATCTCTATTGG | TGTTGGGAGCTCCAGAAATTCTAA |
| wOf53a | TTAACACTTTTAAAACAATGCTACGAAAA | TGATCAAGAAGTTATTGCTGATTTATCTTAT |
| wOf53b | GGATTTCCAACAGAAACCGATGA | TGCGTGTTTTTCCGCTTTGA |
| wOf53c | TGACCTTAGCAGTCCCAGGAGAA | TCCTCCAATTACGCTGACTTCTACA |
| wOf53d | GGAGCAAGTCATAAAAAGTATTTTCTTCAGC | TGATCCAAGAATCGCGACCA |
| wOf54 | TGTCAGTGAGTTTGGTAAACTAAGTTGAATGT | GCTTAAAATGGAACAAAATTGGAGAC |
| wOf55 | TTGCACCTGCAGGAACAGTC | CCTTCTCCCCAGATACTACCTGTAA |
| wOf58 | TCTTTGTTTACCGAAGGTTAAATTTGGA | GCATCGGATATATCTTCTACGCTTGA |
| wOf59 | TGGCATAGGCTTATCTTCTGCAAG | TGCATCTACTTTCTGTGTTGACAATTT |
| wOf61 | TTCCGCTTGGAGATTATGCAAT | TGTGGCTTATAAGAAACCCCGAAA |
| wOf62 | GCTCTTCGCTCAGGGTCTGC | GCACTCTCCGCAGAGACTCTACCT |
| wOf63 | TTTTGGCATCGTGATATTGAGCA | CTATGCAGTTATTGTAGCTCATGATGG |
| wOf64 | CACTTCTGCATGTATGCCGTCAC | GCTTGAAACACGGAACTGTCACTG |
| wOf65 | AAGATTTCCGAATCGAAAGTAATGC | AGATGACGGGAGAGAAGAATAAGATTT |
| wOf66 | TGCTCAGTTGGCTTTGTCTCCA | AGAGCGGAAACTTCGTCTGTCA |
| wOf68a | CCAAGCGAATCTGAGAGAATAGTTGA | GAGTAATGCAACAATGTGATTCATGG |
| wOf68b | GCATTCTGCGACTAGCAAGATAGGA | TGGGAAATAGAACGTCTTTACCTCAAT |
| wOf68c | TGTGGGGCAGTGAAAAGATCC | CAGCCAGCAGTAGCACGTCA |
| wOf69 | AAAAGTTGAGTTGTTAAGGATTTTTATAGCTG | TCGATTTCACCGGCAAATACAT |
| wOf70 | GCGCATCAAGATTAACGCTTTC | TGCGGACGGTATAGCAATGACT |
| wOf71 | GGGTGATTCCGTAAGCACAGA | TTGATTGCAGCATCGATTTGA |
| wOf72 | TCTGAAGATGCATCGCTTGTGA | GATGATAGCGGATATTACCGTTGCT |
| wOf73 | AACAATTTCTTCACACGAAGTTTTGC | CCCGACAAACTAAGAAATCTGCGTAA |
| wOf74 | ACAACCTTTAGGAATTTCACTTGCACA | TAAATTTTACTGCTGCTTTAACTGGTGA |
| wOf75 | TTGGCGGTGTTCAAGATCCA | TGATTTTAGCTCCAATTTTCTCTGCAC |
| wOf76a | CCATGCCATGTAATTTAGGACCA | TCGAGCAAAAGTCAGTATGGATTTC |
| wOf76b | TGTGAGTCATTAGGCGAAGAACTAAA | TGGATCTTGAACACCGCCAAT |
| wOf77 | TGTAAGAGTAGATGCTGATATTCTGCAAG | AACTTCTGGCCAAAGAGAATTTTT |
| wOf78 | TCAATAACTTTCTCACCAGCATATACATAGTT | TTTCATTGTAACCCAAATGCATGA |
| wOf79 | AATTAGATGCAAATTTGATGAAGTTATTGA | TGTTTAGGAGTATGATATCTATTCGCAACTGA |
| wOf80 | TGAGTAATCAAGGCGGAAAACAA | CCGATAATGAGAAAAGTCATTAACCA |
| wOf83 | GAGAAATTCAAAACATTGTTGATATTGAAGA | GCGGCCTGCTTTGAGATGAA |
| wOf84 | TGTTTCGATAGGTATTTTACACGCAAT | TTTATTTGAATGTCAACATTATTGTTCTG |
| wOf86 | AAGAGATGTACTACTTATGGTAAATAAGCTTGAGA | GCAACAGTCTTTTATTGCTGGATGA |
| wOf87 | GCAATAGTGTAGCGCAAAGAAGCA | GGAACACTGTACTTTTAACTTAGATGAATCCAC |
| wOf92 | TCGTTTGGTTTTAAATCTGCAGTAA | AAATGAGAGTAAAAGAACTTTAAATAATACTTTGG |
| wOf94 | GCAGTTCCATTTGCGGAAAAA | TTTGGGTTCTGTTTGCATTATTCAC |
| wOf95a | CCTGCTCATTGTGCTGGTCA | AATGCTGCCTCACAACAGAAGA |
| wOf95b | CCGGTGAAAACACACATAAGAACG | TGTGGGGCTCAAGTTTCTCTATCA |
| 2-MAS | ttgatttaatagtcaatgaaccaaag | acatccgctggcacttgt |
| pol I | tttttccgaaattaacacttttca | tttggttccaaagtttgcagt |
